# The Odyssey of the Ancestral Escherich Strain through Culture Collections: an Example of Allopatric Diversification

**DOI:** 10.1128/mSphere.00553-17

**Published:** 2018-01-31

**Authors:** M. Desroches, G. Royer, D. Roche, M. Mercier-Darty, D. Vallenet, C. Médigue, K. Bastard, C. Rodriguez, O. Clermont, E. Denamur, J.-W. Decousser

**Affiliations:** a Assistance Publique: Hôpitaux de Paris, Hôpital Henri Mondor, Université Paris Est Créteil, Département de Microbiologie, Créteil, France; b UMR1137, Université Paris Diderot, INSERM, IAME, Sorbonne Paris Cité, Paris, France; c CEA DRF Genoscope LABGeM, CNRS UMR8030 Génomique Métabolique, Université d’Evry-Val d’Essonne, Université Paris-Saclay, Evry, France; d Assistance Publique: Hôpitaux de Paris, Hôpital Henri Mondor, Université Paris Est Créteil, Département de Microbiologie, Next-Generation Sequencing Platform, Créteil, France; e Assistance Publique: Hôpitaux de Paris, Hôpital Bichat, Laboratoire de Génétique Moléculaire, Paris, France; University of Wyoming

**Keywords:** *Escherichia coli*, SPANC tradeoff, antibiotic hypersusceptibility, collections, mutator

## Abstract

More than a century ago, Theodor Escherich isolated the bacterium that was to become *Escherichia coli*, one of the most studied organisms. Not long after, the strain began an odyssey and landed in many laboratories across the world. As laboratory culture conditions could be responsible for major changes in bacterial strains, we conducted a genome analysis of isolates of this emblematic strain from different culture collections (England, France, the United States, Germany). Strikingly, many discrepancies between the isolates were observed, as revealed by multilocus sequence typing (MLST), the presence of virulence-associated genes, core genome MLST, and single nucleotide polymorphism/indel analyses. These differences are correlated with the phylogeographic history of the strain and were due to an unprecedented number of mutations in coding DNA repair functions such as mismatch repair (MutL) and oxidized guanine nucleotide pool cleaning (MutT), conferring a specific mutational spectrum and leading to a mutator phenotype. The mutator phenotype was probably acquired during subculturing and corresponded to second-order selection. Furthermore, all of the isolates exhibited hypersusceptibility to antibiotics due to mutations in efflux pump- and porin-encoding genes, as well as a specific mutation in the sigma factor-encoding gene *rpoS*. These defects reflect a self-preservation and nutritional competence tradeoff allowing survival under the starvation conditions imposed by storage. From a clinical point of view, dealing with such mutator strains can lead microbiologists to draw false conclusions about isolate relatedness and may impact therapeutic effectiveness.

**IMPORTANCE** Mutator phenotypes have been described in laboratory-evolved bacteria, as well as in natural isolates. Several genes can be impacted, each of them being associated with a typical mutational spectrum. By studying one of the oldest strains available, the ancestral Escherich strain, we were able to identify its mutator status leading to tremendous genetic diversity among the isolates from various collections and allowing us to reconstruct the phylogeographic history of the strain. This mutator phenotype was probably acquired during the storage of the strain, promoting adaptation to a specific environment. Other mutations in *rpoS* and efflux pump- and porin-encoding genes highlight the acclimatization of the strain through self-preservation and nutritional competence regulation. This strain history can be viewed as unintentional experimental evolution in culture collections all over the word since 1885, mimicking the long-term experimental evolution of *E. coli* of Lenski et al. (O. Tenaillon, J. E. Barrick, N. Ribeck, D. E. Deatherage, J. L. Blanchard, A. Dasgupta, G. C. Wu, S. Wielgoss, S. Cruveiller, C. Médigue, D. Schneider, and R. E. Lenski, Nature 536:165–170, 2016, https://doi.org/10.1038/nature18959) that shares numerous molecular features.

## Abstract (precis)

Mutator phenotypes have been described in laboratory-evolved bacteria, as well as in natural isolates. Several genes can be impacted, each of them being associated with a typical mutational spectrum. By studying one of the oldest strains available, the ancestral Escherich strain, we were able to identify its mutator status leading to tremendous genetic diversity among the isolates from various collections and allowing us to reconstruct the phylogeographic history of the strain. This mutator phenotype was probably acquired during the storage of the strain, promoting adaptation to a specific environment. Other mutations in *rpoS* and efflux pump- and porin-encoding genes highlight the acclimatization of the strain through self-preservation and nutritional competence regulation. This strain history can be viewed as unintentional experimental evolution in culture collections all over the word since 1885, mimicking the long-term experimental evolution of *E. coli* of Lenski et al. (O. Tenaillon, J. E. Barrick, N. Ribeck, D. E. Deatherage, J. L. Blanchard, A. Dasgupta, G. C. Wu, S. Wielgoss, S. Cruveiller, C. Médigue, D. Schneider, and R. E. Lenski, Nature 536:165–170, 2016, https://doi.org/10.1038/nature18959) that shares numerous molecular features.

## INTRODUCTION

*Escherichia coli* is undoubtedly the most studied organism of all time, with many applications in different scientific fields worldwide. Thanks to Theodor Escherich, the first strain was isolated in the late 19th century under the original designation *Bacterium coli commune* (1). Subsequently renamed *E. coli*, the ancestral strain has been deposited in the National Collection of Type Cultures (NCTC) in England as the NCTC86 isolate. Recently, some authors took advantage of the ever-growing importance of next-generation sequencing to study this historical strain (2–4). So, the ancestral Escherich strain was reported to be a commensal strain with no specific resistance trait, belonging to the A phylogroup, closely related to laboratory-derived *E. coli* strain K-12 (2).

After its isolation in 1885 in Munich, Germany, the ancestral Escherich strain began an odyssey in Europe before its inclusion in the English collection in 1920 (4), followed by dissemination to many type culture collections across the world (France, the United States, Germany, Spain, Poland). However, it has been reported that *E. coli*, as well as *Salmonella enterica*, *Bacillus subtilis*, or *Caulobacter crescentus*, can undergo evolution during storage and life in the laboratory (5–13). For example, it has been shown that *rpoS*, which encodes a sigma subunit of RNA polymerase with a major role in stationary phase or under stress conditions (14), can be inactivated under specific storage conditions, such as stab storage (5, 9, 14). Moreover, culture cycles may also have an impact on genes like *mutL*, which encodes the MutL protein involved in DNA mismatch repair (MMR) by assisting MutS in mismatch recognition (9, 15). Such mutations induce a mutator phenotype that may lead to an important divergence between the isolates and their ancestors, highlighting the major effect of strain domestication on genetic content (9).

In this context, we decided to explore the putative genetic evolution of the ancestral Escherich strain through its passage among the main culture collections, as it represents one of the oldest domestication processes of a bacterial strain. To do this, we sequenced the genome of ancestral Escherich strain isolates from four collections (England, France, the United States, and Germany) and compared their genetic and phenotypic characteristics at different scales according to their history. From the historical odyssey of this emblematic strain, we were able to draw some conclusions that could be useful when exploring genetic relatedness between strains by the classical typing methods, as well as by whole-genome sequencing (WGS).

## RESULTS

### Global genetic characterization reveals differences between isolates.

We sequenced the genomes of isolates from the English (NCTC86), French (CIP61.11), American (ATCC 4157), and German (DSM301) collections by using Illumina technology (Table 1). We also retrieved English isolate sequences previously obtained by others (NCTC86_Dunne [Pacific Biosciences and Illumina technologies], NCTC86_Meric [Illumina technology]) (2, 4). First, we focused on multilocus sequence typing (MLST) analysis (16, 17). Using the Warwick scheme, all NCTC86 isolates and CIP61.11 exhibited the same allelic profile corresponding to sequence type 10 (ST10). For its part, ATCC 4157 corresponded to ST7610, differing from ST10 by only one single nucleotide polymorphism (SNP) in allele 8 of *icd*. DSM301 exhibited one more SNP in the same gene, resulting in ST7609. Using the Pasteur scheme, all of the isolates corresponded to ST832, with one SNP in *putP* and *trpB* compared to ST2. As previously described (2), the same serotype, O15:H10, was predicted for all isolates except CIP61.11 for which O type genes were truncated by contig borders. We also analyzed the antimicrobial resistance (AMR) gene content of the isolates by using ResFinder (18). As described by others (2, 4), we did not find any acquired AMR genes (Table 2).

**TABLE 1:** Main characteristics of the ancestral Escherich strain isolates, the technology used for sequencing, and the corresponding references

Isolate	Collection	Country	Yr of entry into collection	Sequencing technology	Reference
NCTC86_Dunne	NCTC	England	1920	Pacific Biosciences RS II, Illumina	2
NCTC86_Meric	NCTC	England	1920	Illumina MiSeq	4
NCTC86	NCTC	England	1920	Illumina MiSeq	This study
CIP61.11	Centre de Ressources Biologiques de l’Institut Pasteur	France	1961	Illumina MiSeq	This study
ATCC 4157	ATCC	United States	Unknown	Illumina MiSeq	This study
DSM301	DSMZ	Germany	1970	Illumina MiSeq	This study

**TABLE 2:** Global-scale genetic characteristics of the ancestral Escherich strain isolates

Isolate	ST Warwick	ST Pasteur	Serotype	Virulence genes^a^	Plasmid replicons
NCTC86_Dunne	10	832	O15:H10	*iss*, *nfaE*, *iha*, *capU*, *gad*, *kpsE*, *hek*, *ompT*, *fyuA*, *irp2*, *malX*	
NCTC86_Meric	10	832	O15:H10	*iss*, *nfaE*, *iha*, *capU*, *gad*, *kpsE*, *hek*, *ompT*, *fyuA*, *irp2*, *malX*	
NCTC86	10	832	O15:H10	*iss*, *nfaE*, *iha*, *capU*, *gad*, *kpsE*, *hek*, *ompT*, *fyuA*, *irp2*, *malX*	
CIP61.11	10	832	O15:H10	*iss*, *nfaE*, *iha*, *capU*, *gad*, *kpsE*, *hek*, *ompT*, *fyuA*, *irp2*, *malX*	
ATCC 4157	7610	832	O15:H10	*iss*, *nfaE*, *iha*, *capU*, *gad*, *kpsE*, *hek*, *ompT*, *fyuA*, *irp2*, *malX*, ***iucC***, ***traT***	IncFIA, IncFII, IncFIB
DSM301	7609	832	O15:H10	*iss*, *nfaE*, *iha*, *capU*, *gad*, *kpsE*, *hek*, *ompT*, *fyuA*, *irp2*, *malX*, ***iucC***, ***traT***	IncFIA, IncFII, IncFIB

a Boldface virulence genes are not shared by all isolates. None of the isolates have acquired AMR genes.

Next, we looked for virulence-associated genes by using VirulenceFinder (19) and the Virulome tool of the MicroScope platform (20). Eleven virulence factors (VFs) were retrieved from all of the isolates (Table 2). Surprisingly, we noticed the additional presence of the siderophore-encoding gene *iucC* and the outer membrane protein-encoding gene *traT* only in ATCC 4157 and DSM301. These two genes are known to be plasmid-derived VFs.

Moreover, PlasmidFinder found three replicon sequences (IncFIA, IncFII, IncFIB) only in ATCC 4157 and DSM301 (Table 2) (21). To confirm that the American and German isolates really contained a plasmid, we compared these two isolates with the English and French ones, searching for specific genes. We found four and five contigs in ATCC 4157 and DSM301, respectively, that can be considered plasmidic. Among the 125 predicted genes on these contigs, we found known plasmidic genes such as *repA/E*, *ccdA*/*B*, and *sopA/B*, in addition to the aerobactin operon *iuc* and the transfer operon *tra* (see Table S1 in the supplemental material). Finally, by using the Plasmid PubMLST sequence definition database, we characterized this plasmid as F1:A6:B1.

10.1128/mSphere.00553-17.2 TABLE S1  Plasmidic genes found in ATCC 4157 and DSM301. Download TABLE S1, PDF file, 0.3 MB. Copyright © 2018 Desroches et al. 2018 Desroches et al. This content is distributed under the terms of the Creative Commons Attribution 4.0 International license.

Thus, this first survey of genetic differences involving tools classically used in WGS epidemiology revealed some surprising differences between isolates.

### The number of mutations correlates with the temporospatial history of the isolates.

**FIG 1:**
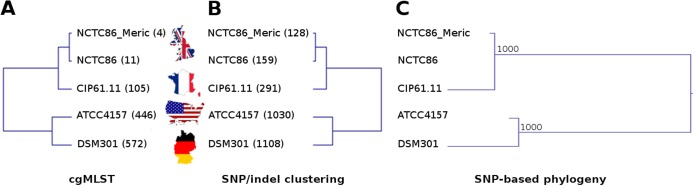
Phylogeography of ancestral Escherich strain isolates. (A) cgMLST-based UPGMA tree. The values in parentheses are numbers of allelic differences from the NCTC86_Dunne strain. (B) Hierarchical clustering (average-linkage Manhattan clustering) tree based on the presence/absence of SNPs and indels. The values in parentheses are numbers of SNP/indel differences from the NCTC86_Dunne strain. (C) Phylogenetic tree based on SNP concatenation by the maximum-likelihood method. Bootstrap values over 1,000 are indicated above the nodes. Maps of the countries of origin of the isolates filled with the respective flags are shown between panels A and B.

To go further in the observed differences, we performed a core genome MLST (cgMLST) comparison of the isolates by using Ridom SeqSphere+ (Ridom GmbH, Münster, Germany) (22). This technique is based only on the coding sequences (CDS) and consists of a gene-by-gene allele calling of core genes. It showed that the isolates differed by many alleles (Fig. 1A), even the English ones. In fact, when comparing NCTC86_Dunne with NCTC86_Meric and NCTC86, we observed 4 and 11 allelic differences, respectively. The French one appeared even more distant, with 105 allelic differences from NCTC86_Dunne. Finally, the American and German ones displayed the largest number of allelic differences from NCTC86_Dunne, 446 and 572, respectively. We also did SNP/indel calling by using the complete genome of NCTC86_Dunne as the reference. The results were in agreement with those of cgMLST (Fig. 1B). After hierarchical clustering of SNP/indel presence/absence in the isolates, we observed two groups: on the one hand, the English/French isolates, with 0/128, 17/142, and 164/127 SNPs/indels in NCTC86_Meric, NCTC86, and CIP61.11, respectively, and on the other hand, the American/German isolates, with 803/227 and 878/230 SNPs/indels in ATCC 4157 and DSM301, respectively. Finally, we performed an SNP-based phylogeny analysis with PhyML (23) (Fig. 1C). The tree obtained was congruent with both previous analyses, with the English and French isolates being relatively close to each other and the American and German ones quite divergent.

We then tried to reconstruct the history of each isolate from the documents supplied by the providers. The strain was transmitted from the NCTC to the Pasteur Institute in 1961 and to the American Type Culture Collection (ATCC) at an unknown date. Finally, the American collection gave it to the German one in April 1970. Assuming that the strain arrived earlier in the ATCC than in the French collection, a correlation was observed between the number of mutations and the temporospatial history of the isolates (Fig. 1), providing a phylogeography of the ancestral Escherich strain in the collections.

### Mutations are randomly distributed across the genome without any trace of selection pressure.

**FIG 2:**
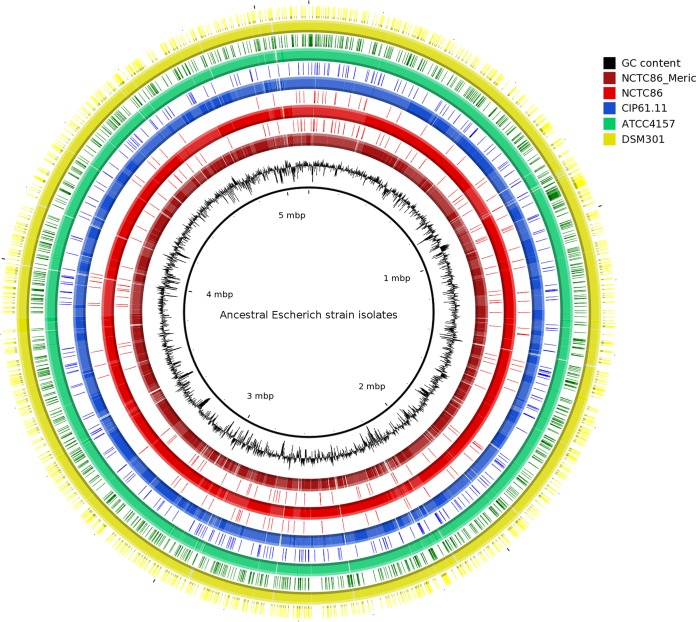
Circular representation of ancestral Escherich strain isolates and physical distribution of SNPs/indels. All isolates are compared to the NCTC86_Dunne genome. From inside to outside, the first circle shows a plot of NCTC86_Dunne GC content. Circles 2 and 3, 4 and 5, 6 and 7, 8 and 9, and 10 and 11 show BLAST comparisons and SNP/indel positions on the genomes of NCTC86_Meric, NCTC86, CIP61.11, ATCC 4157, and DSM301, respectively. Small portions of the genomes are lacking because of unfinished status compared to Pacific Biosciences- and Illumina-sequenced NCTC86_Dunne. The origin corresponds to the threonine operon.

To better understand the origin of these mutations, we depicted the physical distribution of SNPs/indels and did not identify any hot spot of mutations but rather a random distribution of mutations all along the chromosome (Fig. 2) (24). We also compared the function of the CDS affected by mutations on the basis of their Clusters of Orthologous Groups (COGs) classification (Table S2) (25). There was no glaring difference in proportion between the Clusters of Orthologous Groups (COG) profile of the mutated CDS and the COG profile of the NCTC86_Dunne CDS. Finally we estimated the *K*_*a*_/*K*_*s*_ ratio of each isolate and found that it was close to 1 for the four isolates (NCTC86, CIP61.11, ATCC 4157, and DSM301 had *K*_*a*_/*K*_*s*_ ratios of 1.07, 0.94, 1.05, and 1.07, respectively). Because we only found indels in NCTC86_Meric, we were unable to calculate the *K*_*a*_/*K*_*s*_ ratio of that isolate. A ratio of 1 is rather a sign of neutral evolution. All of these results suggest the absence of a selection footprint on the genomes of the isolates.

10.1128/mSphere.00553-17.3 TABLE S2  COG distribution. Download TABLE S2, PDF file, 0.2 MB. Copyright © 2018 Desroches et al. 2018 Desroches et al. This content is distributed under the terms of the Creative Commons Attribution 4.0 International license.

### Mutational spectrum points to the inactivation of several antimutator genes.

**FIG 3:**
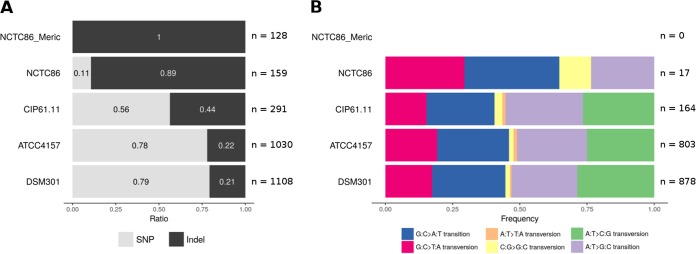
Mutational spectra of the ancestral Escherich strain isolates. (A) Indel/SNP ratios are shown for each isolate compare to NCTC86_Dunne. The total numbers of mutations are indicated to the right of the graph. (B) Mutational spectra of the SNPs of the isolates. Each color represents a possible substitution. The total numbers of SNPs are indicated to the right of the graph.

To gain insight into the mechanisms of mutagenesis, we characterized the mutational spectrum of each isolate (Fig. 3). We first observed a decreasing ratio of indels/substitutions from 1 for NCTC86_Meric to 0.22 and 0.21 for the ATCC and DSM isolates, reflecting the phylogeography of the isolates (Fig. 3A). We then determined the type of mutations and observed in the CIP, ATCC, and DSM isolates roughly a quarter of each type of transition and of the A:T>C:G and G:C>T:A transversions, the NCTC isolate being devoid of A:T>C:G transversions (Fig. 3B).

Taking into account the high level of mutations and differences in their composition, we postulate that these observations could be explained by a mutator phenotype. To confirm that, the sequences of well-known genes conferring a mutator phenotype (e.g., methyl-directed MMR, repair of oxidized guanines) (26) were compared against the ones in the *E. coli* K-12 MG1655 strain isolated from the feces of a convalescent diphtheria patient in Palo Alto in 1922 and known as a nonmutator strain (27). The list of the genes, the observed mutations at the protein level; the prediction of the functional effect on each isolate by SIFT, PolyPhen-2, and PROVEAN softwares (28–30); and their frequency in the UniProt data bank are presented in Table S3. Among the mutated genes, we observed in all isolates a deletion of leucine and alanine (L68_A69del) in a repeated region of MutL that is associated with the mutator phenotype (31, 32). In addition, we found other mutations in the *dam*, *mutS*, and *uvrD* genes that could potentially explain, with that of *mutL*, the elevated rate of indels and transitions that is a typical profile of MMR mutations (26). We also observed in all isolates a C insertion in a tract of C’s in *mutT* resulting in a previously described frameshift (33). When mutated, MutT no longer hydrolyzes the pool of oxidized guanines, leading to a high rate of A:T>C:G transversions (26, 34, 35), which represented roughly 25% of the substitutions observed in the CIP61.11, ATCC 4157, and DSM301 isolates. Finally *miaA* also displayed a probably damaging mutation in ATCC 4157 and DSM301. Such a mutation in the transferase encoded by *miaA* has been implicated in the increase in G:C>T:A transversions (26). Observed mutations in other potential mutator genes were benign or possibly damaging with a lower confidence index and/or not rare in data banks.

10.1128/mSphere.00553-17.4 TABLE S3  Mutator genes, mutations, and predictions of their effects. Download TABLE S3, PDF file, 0.2 MB. Copyright © 2018 Desroches et al. 2018 Desroches et al. This content is distributed under the terms of the Creative Commons Attribution 4.0 International license.

We then measured the capacity to generate mutations in the *rpoB* gene conferring resistance to rifampin (Table 3), a classical assay to evaluate the mutation rate in bacteria (36, 37). The four isolates NCTC86, CIP61.11, ATCC 4157, and DSM301 exhibited a significantly higher mutation rate than the nonmutator control *E. coli* CIP2.83 strain (*E. coli* W) isolated around 1943 from the soil of a cemetery near Rutgers University (38), ranging from 2.11 ⋅ 10^−7^ to 1.28 ⋅ 10^−6^, in the range of what has been reported in natural isolate MMR mutants (37).

**TABLE 3:** Phenotypic characteristics of the ancestral Escherich strain isolates

**Isolate**	Mutation rate^a^	Mouse model of extraintestinal virulence^b^	Antimicrobial susceptibility profile^c^
NCTC86	4.38 ⋅ 10^−7^	0/10	S^d^
CIP61.11	1.28 ⋅ 10^−6^	0/10	S^e^
ATCC 4157	8.29 ⋅ 10^−7^	0/10	S^f^
DSM301	2.11 ⋅ 10^−7^	0/10	S^d^

a Mutation rates correspond to median values of five independent experiments, with *E. coli* CIP2.83 and M13 being used as nonmutator (mutation rate, 7.80 ⋅ 10^−9^) and MMR-deficient (mutation rate, 1.32 ⋅ 10^−7^) control strains, respectively.

b The number of mice killed by the isolate/number of mice inoculated is shown. CFT073 was used as the killer control strain (10/10), and K-12 MG1655 was the nonkiller control strain (0/10).

c S, susceptibility to all of the antibiotics tested except erythromycin.

d Hypersusceptible to ampicillin, piperacillin, ticarcillin-clavulanic acid, cephalothin, cefixime, cefotaxime, aztreonam, chloramphenicol, tetracycline, nalidixic acid, levofloxacin, ofloxacin, ciprofloxacin, norfloxacin, moxifloxacin, gentamicin, and co-trimoxazole.

e Hypersusceptible to amoxicillin, ampicillin, piperacillin, ticarcillin-clavulanic acid, cephalothin, cefixime, cefotaxime, aztreonam, chloramphenicol, tetracycline, colistin, nalidixic acid, levofloxacin, ofloxacin, ciprofloxacin, norfloxacin, moxifloxacin, streptomycin, gentamicin, and co-trimoxazole.

f Hypersusceptible to ampicillin, piperacillin, ticarcillin-clavulanic acid, cefixime, cefotaxime, aztreonam, chloramphenicol, tetracycline, nalidixic acid, levofloxacin, ofloxacin, ciprofloxacin, norfloxacin, moxifloxacin, streptomycin, gentamicin, and co-trimoxazole.

These results confirmed the mutator status of the isolates with a never reported high level of antimutator gene inactivation and a very specific mutational spectrum.

### The clinically relevant phenotypes of the mutator status.

We then wanted to assess the effect of the mutator status of the isolates on two phenotypes of clinical importance, i.e., intrinsic extraintestinal virulence and sensitivity to antibiotics. We first assessed if the isolates exhibited a particular virulence phenotype owing, on the one hand, to the modification of the virulence reported for the mutator strains (39) and, on the other hand, to their differences in plasmid content. We used a mouse model of sepsis recording deaths (40) and did not observe any deaths among animals caused by any of our isolates compared to the 100% mortality caused by archetypal extraintestinal pathogenic *E. coli* strain CFT073 (Table 3). We concluded that the Escherich strain isolates were devoid of extraintestinal virulence, in agreement with their belonging to phylogroup A (40), even with the presence of plasmid-related virulence genes in ATCC 4157 and DSM301.

It has been reported that after 1,000 generations, mutator isolates have altered fitness, with a loss of numerous functions such as metabolic pathways, chemical stress, and phage resistance (41). Alternatively, a high mutation rate has been shown to be associated with antibiotic resistance (42, 43). We thus studied the antimicrobial susceptibility of the isolates by estimating the MICs of main antibiotics of clinical interest by the E test method. Every isolate was susceptible to all of the drugs tested according to the Antimicrobial Committee of the French Society of Microbiology-European Committee on Antimicrobial Susceptibility Testing (CASFM-EUCAST), except erythromycin, which is known to be inactive against members of the family *Enterobacteriaceae*. All other MICs were very low, and all of the isolates were hypersusceptible or very susceptible (see Materials and Methods for the definition) to the following antibiotics: β-lactams, chloramphenicol, tetracycline, colistin, quinolones, aminoglycosides, and co-trimoxazole (Table 3; Table S4). It should be noted that we observed a strikingly low MIC of benzylpenicillin, although no cutoff value is available for this antibiotic (Table S4).

10.1128/mSphere.00553-17.5 TABLE S4  Antibiotic susceptibility testing of ancestral Escherich strain isolates. Download TABLE S4, PDF file, 0.3 MB. Copyright © 2018 Desroches et al. 2018 Desroches et al. This content is distributed under the terms of the Creative Commons Attribution 4.0 International license.

We then searched for a genetic basis for these high-susceptibility phenotypes. We looked for mutations in genes involved in efflux because of the wide range of targets of such systems (44), as well as genes encoding the main porins OmpA, OmpF, and OmpC and their regulators EnvZ/OmpR (45). Among them, we found in all of the isolates the same nonsynonymous mutations in five genes (*acrB*, *acrE*, *acrS*, *rob*, and *ompF*), which were predicted to be deleterious (Table S5). Of note, the mutation found in OmpF (E139G, corresponding to E117G without the signal peptide) is located in the channel constriction of the porin (46). Some mutations in this loop have been associated with increased antibiotic susceptibility (47).

10.1128/mSphere.00553-17.6 TABLE S5  Candidate genes related to high susceptibility. Download TABLE S5, PDF file, 0.2 MB. Copyright © 2018 Desroches et al. 2018 Desroches et al. This content is distributed under the terms of the Creative Commons Attribution 4.0 International license.

Thus, we hypothesized that the mutator phenotype rendered the isolates highly susceptible to many antibiotics, presumably because of defects in efflux pumps and porins.

### Squatter colony analysis: an example of convergence with a potential clinical impact.

**FIG 4:**
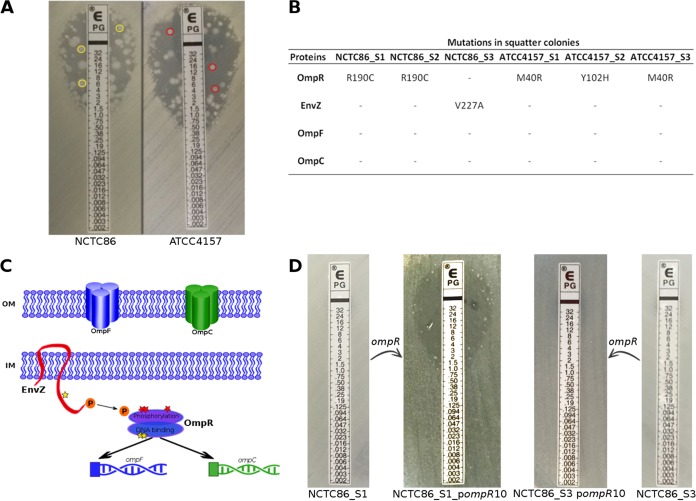
Squatter colonies and mutations in the EnvZ/OmpR system. (A) Squatter colonies of NCTC86 and ATCC 4157 isolates in the growth inhibition zone of the benzylpenicillin E test. Three colonies of each isolate were sequenced (yellow and red circles, respectively). (B) Nonsynonymous mutations in OmpR, EnvZ, OmpF, and OmpC found in the squatter colonies compared to NCTC86 and ATCC 4157. (C) Schematic representation of the EnvZ/OmpR system showing the mutations indicated by a star (yellow and red for the NCTC86 and ATCC 4157 isolates, respectively). OM, outer membrane; IM, inner membrane. (D) Benzylpenicillin E tests of NCTC86_S1, NCTC86_S3, NCTC86_S1/p*ompR*10, and NCTC86_S3/p*ompR*10 (complemented isolates were tested on plates containing kanamycin). The absence of inhibition zone corresponds to a MIC > 32 mg/liter. Note that the sensitive phenotype with some squatter colonies is restored by p*ompR*10 complementation only in the NCTC86_S1 isolate.

We observed colonies of all of the isolates tested inside the growth inhibition zone of many β-lactam (Fig. 4A) and fluoroquinolone antibiotics (Table S4). Such colonies, called squatter colonies, are a classical feature of natural *E. coli* isolates having a mutator phenotype (36). We further studied three randomly selected colonies of the NCTC86 (NCTC86_S1, NCTC86_S2, and NCTC86_S3) and ATCC 4157 (ATCC 4157_S1, ATCC 4157_S2, and ATCC 4157_S3) isolates in the growth inhibition zone of benzylpenicillin, as the phenomenon was more prevalent with this antibiotic and as these strains represent the British-French and American-German groups, respectively. Antibiotic susceptibility testing revealed that these squatter colonies effectively exhibited a high benzylpenicillin MIC (Table S8). They remained susceptible to all of the other drugs tested, with only a few MIC variations from the parental isolates.

We then sequenced these resistant strains to find SNPs that could explain the phenotype observed. To find candidate genes, we focused on indels in CDS and nonsynonymous or nonsense SNPs (Table S6) that were present in squatter sequences but absent from the parental isolate. Among these mutations, we found five genes that were affected in all or nearly all (5/6) of the isolates: the two-component system response regulator *ompR*, a malate transporter, an IS*4* family transposase, and two hypothetical proteins. We further explored only *ompR*, as it appeared to be the most likely presumption, even if it was not recovered from NCTC86_S3 (Fig. 4B). All of the mutations observed in this regulator were predicted to be damaging (Table S7). NCTC86_S1 and NCTC86_S2 had the same mutation at the protein level (R190C) compared to *E. coli* K-12 MG1655. On a homology model of OmpR structure, this amino acid, exposed at the surface of the protein, is positioned at the end of the α2 helix in the helix-turn-helix, a substructure of the C-terminal domain known to be involved in DNA binding (48, 49) (Fig. 4C; Fig. S1). ATCC 4157_S1 and ATCC 4157_S3 exhibited an M40R mutation, which is located in the N-terminal phosphorylation region of the protein (48, 50). Methionine 40 is close in space to the phosphorylation site (Asp 55). Replacing this methionine with an arginine would result in steric hindrance between the α2 and α3 helices. It has also been shown in a previous study that another residue mutation at position 40 can affect OmpR function (51). For ATCC 4157_S2, we highlighted a Y102H substitution. Tyrosine 102 is essential in OmpR because it is part of the hinge region enabling the flexibility of the protein (50) (Fig. S1). As OmpR is part of a two-component system that includes the sensor EnvZ, we also investigated mutations occurring in this protein. Only NCTC86_S3 presented a substitution, V227A (Fig. 4B), which is located in the A domain, which is responsible for dimerization and contains the H box (52). It was predicted to affect protein function (Table S7). However, the three-dimensional model of the protein did not reveal any important modification due to this mutation.

10.1128/mSphere.00553-17.1 FIG S1  Three-dimensional model of the OmpR protein. The model was obtained by homology modeling using as the template structure the response regulator RegX3 from *Mycobacterium tuberculosis* (PDP code 2OQR). Mutations appeared in essential regions of the protein (residues are pink): methionine 40 close to the phosphorylation site (Asp 55), tyrosine 102 in the hinge region, and arginine 190 in the helix-turn-helix-like structure. Download FIG S1, TIF file, 2.7 MB. Copyright © 2018 Desroches et al. 2018 Desroches et al. This content is distributed under the terms of the Creative Commons Attribution 4.0 International license.

10.1128/mSphere.00553-17.7 TABLE S6  Candidate genes related to resistance in squatter colonies. Download TABLE S6, PDF file, 0.3 MB. Copyright © 2018 Desroches et al. 2018 Desroches et al. This content is distributed under the terms of the Creative Commons Attribution 4.0 International license.

10.1128/mSphere.00553-17.8 TABLE S7  OmpR and EnvZ mutations and predictions of their effects. Download TABLE S7, PDF file, 0.2 MB. Copyright © 2018 Desroches et al. 2018 Desroches et al. This content is distributed under the terms of the Creative Commons Attribution 4.0 International license.

10.1128/mSphere.00553-17.9 TABLE S8  Antibiotic susceptibility testing of squatter colonies. Download TABLE S8, PDF file, 0.1 MB. Copyright © 2018 Desroches et al. 2018 Desroches et al. This content is distributed under the terms of the Creative Commons Attribution 4.0 International license.

To definitively implicate the *ompR* mutations in benzylpenicillin resistance, we complemented the six squatter isolates with plasmid-borne *ompR* as described in reference 53 and restored hypersusceptibility to benzylpenicillin in all of the isolates except NCTC86_S3 (Fig. 4D).

All of these results showed an evolutionary convergence of the squatter colonies which all mutated in different parts of this two-component system, leading to increased MICs of benzylpenicillin. Interestingly, it has been shown that mutations in this gene can also confer resistance to ertapenem in an extended-spectrum-β-lactamase-producing *E. coli* isolate in a clinical context (53).

## DISCUSSION

The genome of the ancestral Escherich strain has recently been reported in the literature (2–4), and this strain can be considered one of the oldest bacterial strains available. As genetic changes in strains during conservation and domestication have previously been reported (5, 9), we used this emblematic bacterial strain to explore the genomic drift that occurs in different type culture collections.

The most striking feature of our work is that we found major differences between the different isolates at every level of granularity of our analysis and that these differences are mostly due to the ancestral Escherich strain’s mutator status, due to multiple mutations, including genes of the MMR system and the repair of oxidized guanines (Table S3). Among them, we observed previously described mutations in *mutL* and *mutT* responsible for the strong mutator phenotype (32, 33) and others in *dam*, *mutS*, and *uvrD*. We also found a probably damaging mutation in the transferase-encoding gene *miaA*, but only in ATCC 4157 and DSM301. Mutations in this gene have been shown to increase the mutation frequency 6- to 30-fold, especially G:C>T:A transversions (54). The amino acid changes found in *mutY* and *mutM* are polymorphisms rather than deleterious mutations, so it is unlikely that they bring an antimutator effect to counterbalance the impact of *mutT* mutation, as previously described (55, 56). Our unique data set allows us to see the sequential impact on the mutational spectra of the mutator genes with first the effect of the inactivation of the MMR (indels and transitions) and then the effect of MutT inactivation (A:T>C:G transversions) when SNPs are increasing (Fig. 3). The presence of G:C>T:A transversions is more difficult to interpret but probably reflects oxidative stress. Interestingly, when we phenotypically measured the *rpoB* gene mutation rate in the isolates (Table 3), we found values in the range of what has been observed in MMR mutants (57).

This raises the question of the timing of strain mutator status emergence. Mutators are frequently encountered in natural isolates, sometimes reaching >1% of the population, making it plausible that what Escherich isolated in 1885 was a mutator strain (37, 58). But laboratory culture conditions could also be responsible for such mutations, as described by Liu et al. (9). The latter hypothesis is reinforced by the fact that we observed in all of the isolates a mutation in *rpoS* leading to a deletion of the last amino acid with the addition of a SICQKG tail, similar to the addition of 39 amino acids at the C-terminal end of RpoS reported after 10 days of *in vitro* evolution in stationary phase (growth advantage in stationary-phase cells) (59). Such a mutation in *rpoS* is the expression of self-preservation and nutritional competence (SPANC) balance regulation (60, 61). Indeed, during their conservation, the strains may trade their general stress resistance against improved metabolism to allow survival under the starvation conditions imposed by storage. It also probably explains the mutation in OmpF, another pivotal system in SPANC balance (60), which enables a larger input of substrates in the antibiotic-free environment of stab storage. By using the *E. coli* collection of reference strains, it has been shown that mutations affecting porin proteins are cornerstones of the inverse relationship between competitiveness and the resistance of strains to antibiotics (62). The Escherich strain has undergone numerous subcultures on various media during its European trip to the Lister Institute (4) and then was preserved at the NCTC in solid agar medium before being freeze-dried in the late 1940s. Shipment to other collections represents additional subcultures selecting such phenomena. However, as soon as the antibiotic pressure reappears, for example, during MIC testing, mutations in EnvZ and OmpR are selected to regulate the porins and thus to tip the balance to the self-preservation side, resulting in increased resistance to benzylpenicillin in squatter colonies. Thus, presumably, the ancestral Escherich strain was not a mutator strain but selected mutator alleles during its conservation in collections, allowing it to cope with life under such conditions by second-order selection (63). It parallels the long-term evolution experiment done by Lenski and colleagues with 12 populations of *E. coli* B (64, 65) across 50,000 generations (66) where 6 populations evolved a hypermutable phenotype, 4 of them exhibiting the *mutL* and *mutT* defects that we observed in the Escherich strain. It also represents a very nice model of allopatric diversification owing to the geographic separation of the isolates linked to the mutator status.

From a more clinical and epidemiological point of view, such mutator isolates can also be problematic when cross contamination between patients is suspected. Indeed, the current gold standard used to compare strains during a putative epidemic event is determination of their cgMLST profiles and analysis of their allelic variations (67, 68). This approach is robust, and its reproducibility has been recently confirmed (69). Mellmann et al. proposed a threshold of 10 differing alleles to discard nosocomial transmission (70). This threshold could be corrupted when mutator strains are involved in an epidemic event. The issue has already been addressed on the SNP calling scale during a *Staphylococcus aureus* outbreak in the United Kingdom; among the epidemic isolates, the investigators identified a divergent one in terms of SNP numbers relating to the mutator phenotype (71). In our case, we will have drawn a false conclusion by considering that each isolate is different while they all belong to the same strain, even when looking only at the less sensitive MLST results. Storage and subculturing of strains in collections also lead to plasmid loss, as reported, for example, for the archetypal enteropathogenic *E. coli* E2348/69 strains (72). Although we did not identify any difference in the mouse virulence assay between the isolates exhibiting or not exhibiting the plasmid (Tables 2 and 3), such plasmid content differences can impact the virulence and/or resistance of isolates.

From a therapeutic point of view, it has been shown that mutators can more easily acquire antibiotic resistance through mutations and high rates of recombination (57). The antimicrobial susceptibility profile of our isolates was typical of mutator strains with the presence of squatter colonies (36). By studying these squatter colonies, we were able to highlight a convergence on the two-component system response regulator EnvZ-OmpR leading to β-lactam resistance. The same kind of mutation has previously been reported and led to the emergence of resistance to a broad-spectrum antibiotic such as ertapenem (53).

In conclusion, through the odyssey of the ancestral Escherich strain isolates, we were able to measure the effect of a mutator strain in culture collections. It pointed out the risk of misinterpretation of several phenotypes such as antibiotic susceptibility, as well as the epidemiological relatedness of strains. Therefore, interpretation of WGS data should trigger a thorough analysis of antimutator genes.

## MATERIALS AND METHODS

### Ancestral Escherich strain isolates, culture conditions, and DNA extraction.

Isolates from four national collections were recovered from freeze-dried or lyophilized stocks, i.e., NCTC86 from the English NCTC, CIP61.11 from the collection of the French Pasteur Institute, ATCC 4157 from the ATCC, and DSM301 from the Deutsche Sammlung von Mikroorganismen und Zellkulturen (DSMZ) at the Leibniz Institute. Stocks were rehydrated with brain heart infusion medium for 30 min and then plated on chocolate agar plates. DNA was extracted from a few colonies with the DSP DNA minikit on the QIAsymphony instrument (Qiagen, Hilden, Germany) in accordance with the manufacturer’s instructions. Stocks were prepared from the mass of colonies and stored with glycerol at −80°C.

### Genome sequencing and annotation.

Genomes were sequenced on Illumina MiSeq (read length, 2 × 300 bp; MiSeq Reagent kit v3) after NextEra XT library preparation (Illumina, San Diego, CA). The fastq files were quality trimmed at the ends until an average base quality of 30 was reached in a window of 20 bases. *De novo* assembly was then performed with the Velvet assembler integrated in the SeqSphere+ software (Ridom GmbH, Münster, Germany) with optimized k-mer size (22). The fasta files obtained were then annotated on the MicroScope platform (http://www.genoscope.cns.fr/agc/microscope). As NCTC86 isolates were already sequenced on Illumina MiSeq by Meric et al. (4) and on the Pacific Biosciences RS II instrument and Illumina by Dunne et al. (2), we downloaded the corresponding GenBank files (GenBank accession no. MCAV00000000.1 and NZ_LT601384.1). These genomes were also integrated and reannotated on the same platform. The COG functional categories of the predicted proteins were determined with the COGNiTOR tool (25).

### Genetic characterization.

Each isolate was genetically characterized with ResFinder 2.1 (18), VirulenceFinder 1.5 (19), PlasmidFinder 1.3 (DB-Enterobacteriaceae) (21), MLST 1.8 (73), and SerotypeFinder 1.1 (74) by using the default threshold. We also looked for extraintestinal VFs with the Virulome tool on the MicroScope platform by using an identity threshold of ≥80% (20). To retrieve plasmidic contigs and their annotation, we searched for homologous genes present in DSM301 and ATCC 4157 but absent from NCTC86_Dunne, NCTC86_Meric, NCTC86, and CIP61.11 by using an identity threshold of ≥80% and a minimum coverage of ≥80%. Plasmid typing (MLST) was performed at the PubMLST website (http://www.pubmlst.org/plasmid/) (75).

### cgMLST.

A cgMLST *ad hoc* scheme was constructed with SeqSphere+ with the NCTC86_Dunne genome as the reference by using standard parameters and NCTC86_Meric, NCTC86, CIP61.11, ATCC 4157, and DSM301 as the query genomes (22). The NCTC86_Dunne sequence was used as the reference genome because (i) it corresponds to a complete and circularized chromosome and (ii) the NCTC86 isolate corresponds to the first deposited isolate. Of the 3,888 cgMLST targets obtained, 3,800 were present in all of the genomes. An allele was assigned to each different sequence for a given gene. The number of differences between genomes was then calculated and used to generate a tree by the unweighted pair group method using average linkages (UPGMA).

### SNP/indel calling analysis.

SNP/indel calling was performed with the PALOMA bioinformatic pipeline implemented with the MicroScope platform (20) after a quality trimming step. Forward reads from each sequenced isolate and from NCTC86_Meric were then mapped onto the NCTC86_Dunne sequence. Only unique matches having an alignment score equal to at least half of their length were retained as seeds for full Smith-Waterman realignment (76) with a region of the reference genome extended five nucleotides on both ends. SNPs/indels were filtered on the basis of their coverage (relative coverage of >0.5 base and absolute coverage of >10 bases with a *Q* score of ≥23).

### Phylogenetic analysis.

We concatenated all of the positions where at least one SNP can be found for NCTC86_Meric, NCTC86, CIP61.11, ATCC 4157, and DSM301. We then performed a maximum-likelihood phylogeny with bootstrap support estimation by using PhyML (23) and the GTR-gamma model.

### *K*_*a*_/*K*_*s*_ ratio estimation.

We determined the *K*_*a*_/*K*_*s*_ ratio as previously described (77). Briefly, we estimated the expected number of mutations in NCTC86_Dunne. We then compared this number to the observed number of mutations in CDS, taking into account the type of mutation (transitions or transversions) and the consequence for the protein (nonsynonymous versus synonymous). Nonsense mutations were excluded.

### Prediction of functional effect of nonsynonymous mutations and protein modeling.

We predicted the functional effect of mutations by using the SIFT, PolyPhen-2, and PROVEAN software (28–30). For each protein, we searched available sequences in the UniProt database by using the gene name and focusing on the genus *Escherichia*. We then aligned these sequences with ClustalW and counted the occurrences of the observed alleles (78). We considered a mutation potentially damaging if a minimum of two of these softwares predicted it (a SIFT score of <0.05, a PolyPhen-2 conclusion equal to “probably damaging” or “possibly damaging,” and a PROVEAN score of ≤2.5) and if the mutation was not observed in the database.

Three-dimensional models of the structures of OmpR and EnvZ were built by homology modeling with MODELLER (79) by using as templates the crystallographic structures of, respectively, the response regulator RegX3 from *Mycobacterium tuberculosis* (PDB code 2OQR) (80) and a chimeric histidine kinase of *E. coli* and *Thermotoga maritima* (PDB code 4KP4) (81, 82). The N-terminal domain (residues 1 to 149) of EnvZ was not modeled, as the mutation of interest in EnvZ is positioned in the C-terminal domain. The quality of the homology models of OmpR and of the C-terminal domain of EnvZ was assessed with PROSA-II (https://prosa.services.came.sbg.ac.at/prosa.php) with *Z* scores of −6.5 and −4.82, respectively. Model structures were visualized with PyMol (https://pymol.org/2/).

### Estimation of mutation rates by Rif test.

The mutation rates of the strains were estimated by monitoring the capacity of the strains to generate nonlethal mutations in the *rpoB* gene conferring resistance to rifampin, as described previously (36, 83). *E. coli* CIP2.83 (W) was used as a nonmutator control strain, whereas strain M13 was used as an MMR-deficient (*mutS* large deletion) control strain. Five independent experiments were performed, and the median value is presented.

### Mouse lethality assay.

A mouse model of sepsis following the subcutaneous injection of bacteria was used as previously described (40). Each experimental series included 10 animals per isolate, and virulent (CFT073) and nonvirulent (K-12 MG1655) control strains killing all of the mice or no mice, respectively, were used in each experiment. Animal experiments were carried out in accordance with authorization number 6665 given by the Ministère de l’agriculture, France. The mouse septicemia model was conducted in accordance with European and national regulations for the housing and care of laboratory animals after pertinent review and approval by the Bioethics Committee at Santiago de Compostela University and by the French Veterinary Services (certificate number A75-18-05). All possible measures were taken to minimize animal suffering and to ensure animal welfare. When necessary, animals were sacrificed by lethal intraperitoneal injection of phenobarbital.

### Antimicrobial phenotypic susceptibility.

The antimicrobial susceptibility of each isolate was tested by MIC determination by the E test method (BioMérieux, Craponne, France) in accordance with the manufacturer’s instructions. Results were interpreted as recommended by CASFM-EUCAST (V1.0 2017/03). The antimicrobial compounds tested are listed in Table S4. Using the MIC distributions for *E. coli* provided by EUCAST, we defined hypersusceptible and very susceptible breakpoints as the lowest MICs encompassing 1 and 5% of the strains, respectively.

### Squatter colony complementation and antimicrobial susceptibility testing.

Plasmid p*ompR*10 (53), harboring the wild-type *ompR* gene and the kanamycin resistance gene, was electroporated into cells from the six squatter colonies (NTC86_S1, NCTC86_S2, NCTC86_S3, ATCC 4157_S1, ATCC 4157_S2, and ATCC 4157_S3). Recombinant clones were selected on lysogeny broth agar plates with kanamycin (30 mg/liter), and MIC determinations were performed on Mueller-Hinton agar plates with kanamycin (30 mg/liter) and interpreted as described above.

### Accession number(s).

The annotated genomes of all of the isolates have been deposited in the EMBL database under BioProject accession numbers PRJEB23212 to PRJEB23221.
