# Supplementary material for: The Odyssey of the Ancestral Escherich Strain through Culture Collections: an Example of Allopatric Diversification
Source: mSphere. 2018 Jan 31;3(1):e00553-17. doi: 10.1128/mSphere.00553-17 (PMC5793043; doi:10.1128/mSphere.00553-17)
Supplement: TABLE S1 [file sph001182464st1.pdf]

TABLE S1. Plasmidic genes found in ATCC4157 and DSM301 ancestral Escherich strain isolates with MicroScope platform annotation

Gene label ATCC4157*	Gene label DSM301*	Type	Gene_name	Product
AT4157R_v1_490011	DSM301R_v1_600021	CDS		protein of unknown function
AT4157R_v1_490074	DSM301R_v1_1390004	CDS		ProQ/FINO family protein (fragment)
AT4157R_v1_490048	DSM301R_v1_1390030	CDS		conserved protein of unknown function
AT4157R_v1_2030001	DSM301R_v1_600001	CDS		conserved protein of unknown function
AT4157R_v1_970025	DSM301R_v1_350002	CDS		conserved protein of unknown function
AT4157R_v1_490027	DSM301R_v1_600005	CDS		conserved protein of unknown function
AT4157R_v1_970009	DSM301R_v1_820009	fCDS	ydfJ	fragment of putative transporter (part 2)
AT4157R_v1_490075	DSM301R_v1_1390003	CDS		conserved protein of unknown function
AT4157R_v1_310006	DSM301R_v1_180008	CDS		conserved protein of unknown function
AT4157R_v1_490014	DSM301R_v1_600018	CDS	ccdA	Antitoxin CcdA
AT4157R_v1_490022	DSM301R_v1_600010	CDS		conserved protein of unknown function
AT4157R_v1_490047	DSM301R_v1_1390031	CDS	traR	Protein TraR
AT4157R_v1_970017	DSM301R_v1_820017	CDS		conserved protein of unknown function
AT4157R_v1_970043	DSM301R_v1_350020	CDS		conserved protein of unknown function
AT4157R_v1_490038	DSM301R_v1_1390040	CDS	traY	Relaxosome protein TraY
AT4157R_v1_2030004	DSM301R_v1_600004	CDS		conserved protein of unknown function
AT4157R_v1_490004	DSM301R_v1_600024	CDS	vagC	Virulence-associated protein VagC
AT4157R_v1_490007	DSM301R_v1_600027	CDS	vagC	Virulence-associated protein VagC
AT4157R_v1_490029	DSM301R_v1_1390049	CDS	ykfF	hypothetical protein; CP4-6 prophage
AT4157R_v1_970035	DSM301R_v1_350012	CDS		conserved protein of unknown function
AT4157R_v1_970044	DSM301R_v1_350021	CDS		Protein impB (fragment)
AT4157R_v1_490076	DSM301R_v1_1390002	CDS	repA2	Replication regulatory protein repA2
AT4157R_v1_490056	DSM301R_v1_1390022	CDS	trbE	Protein TrbE
AT4157R_v1_970001	DSM301R_v1_820001	CDS		conserved protein of unknown function
AT4157R_v1_970037	DSM301R_v1_350014	CDS		conserved protein of unknown function
AT4157R_v1_970002	DSM301R_v1_820002	CDS		YacB
AT4157R_v1_490059	DSM301R_v1_1390019	CDS	traQ	Protein TraQ
AT4157R_v1_970036	DSM301R_v1_350013	CDS		conserved protein of unknown function
AT4157R_v1_490033	DSM301R_v1_1390045	CDS		conserved protein of unknown function
AT4157R_v1_490061	DSM301R_v1_1390017	CDS	trbJ	Protein TrbJ
AT4157R_v1_970026	DSM301R_v1_350003	CDS		transposase
AT4157R_v1_970038	DSM301R_v1_350015	CDS		Uncharacterized 11.1 kDa protein
AT4157R_v1_490070	DSM301R_v1_1390008	CDS		TrwC relaxase family protein
AT4157R_v1_490015	DSM301R_v1_1390025	CDS	ccdB	Toxin CcdB
AT4157R_v1_490053	DSM301R_v1_600017	CDS		conserved protein of unknown function
AT4157R_v1_490040	DSM301R_v1_1390038	CDS	traL	Protein TraL
AT4157R_v1_490058	DSM301R_v1_1390020	CDS	trbA	Protein TrbA
AT4157R_v1_490045	DSM301R_v1_1390033	CDS		Conjugal transfer protein TrbD
AT4157R_v1_490039	DSM301R_v1_1390039	CDS	traA	Pilin
AT4157R_v1_310007	DSM301R_v1_180007	CDS	crcB	conserved hypothetical protein; putative inner membrane protein associated with chromosome condensation
AT4157R_v1_970032	DSM301R_v1_350009	CDS		RutC family protein in vnfA 5'region
AT4157R_v1_490036	DSM301R_v1_1390042	CDS	traM	Relaxosome protein TraM
AT4157R_v1_490050	DSM301R_v1_1390028	CDS	trbI	Protein TrbI
AT4157R_v1_490062	DSM301R_v1_1390016	CDS		Conjugal transfer protein TrbF
AT4157R_v1_490003	DSM301R_v1_600025	CDS	vapC	tRNA(fMet)-specific endonuclease VapC
AT4157R_v1_490006	DSM301R_v1_600028	CDS	vapC	tRNA(fMet)-specific endonuclease VapC
AT4157R_v1_490026	DSM301R_v1_600006	CDS		conserved protein of unknown function
AT4157R_v1_490025	DSM301R_v1_600007	CDS	yubI	putative antirestriction protein YubI
AT4157R_v1_310005	DSM301R_v1_180009	CDS		conserved protein of unknown function
AT4157R_v1_490023	DSM301R_v1_600009	CDS		conserved protein of unknown function
AT4157R_v1_490031	DSM301R_v1_1390047	CDS	psiB	Protein PsiB
AT4157R_v1_490073	DSM301R_v1_1390005	CDS		Fertility inhibition protein (fragment)
AT4157R_v1_490066	DSM301R_v1_1390012	CDS	traS	TraS protein
AT4157R_v1_970004	DSM301R_v1_820004	CDS		conserved protein of unknown function
AT4157R_v1_490009	DSM301R_v1_600023	CDS		conserved protein of unknown function
AT4157R_v1_490035	DSM301R_v1_1390043	CDS	X	X polypeptide
AT4157R_v1_490013	DSM301R_v1_600019	CDS		conserved protein of unknown function
AT4157R_v1_490046	DSM301R_v1_1390032	CDS	traV	Protein TraV
AT4157R_v1_490063	DSM301R_v1_1390015	CDS		Protein TraH (fragment)
AT4157R_v1_490028	DSM301R_v1_1390050	CDS	ssb	Single-stranded DNA-binding protein
AT4157R_v1_490060	DSM301R_v1_1390018	CDS	trbB	Protein TrbB
AT4157R_v1_2030002	DSM301R_v1_600002	CDS		conserved protein of unknown function
AT4157R_v1_490041	DSM301R_v1_1390037	CDS	traE	Protein TraE
AT4157R_v1_490044	DSM301R_v1_1390034	CDS	traP	Protein TraP
AT4157R_v1_490068	DSM301R_v1_1390010	CDS		conserved protein of unknown function
AT4157R_v1_490051	DSM301R_v1_1390027	CDS	traW	Protein TraW
AT4157R_v1_490054	DSM301R_v1_1390024	CDS	trbC	Periplasmic protein TrbC
AT4157R_v1_490037	DSM301R_v1_1390041	CDS	traJ	Protein TraJ
AT4157R_v1_490021	DSM301R_v1_600011	CDS	yubD	putative methylase YubD
AT4157R_v1_970006	DSM301R_v1_820006	CDS		conserved membrane protein of unknown function
AT4157R_v1_490032	DSM301R_v1_1390046	CDS	psiA	Protein PsiA
AT4157R_v1_490042	DSM301R_v1_1390036	CDS	traK	TraK lipoprotein
AT4157R_v1_490067	DSM301R_v1_1390011	CDS	traT	TraT complement resistance protein
AT4157R_v1_490064	DSM301R_v1_1390014	CDS		Protein TraH (fragment)
AT4157R_v1_970041	DSM301R_v1_350018	CDS	intM	putative site-specific recombinase
AT4157R_v1_490057	DSM301R_v1_1390021	CDS	traF	Protein TraF
AT4157R_v1_490072	DSM301R_v1_1390006	CDS	traX	Protein TraX
AT4157R_v1_970034	DSM301R_v1_350011	CDS	rhmR	Uncharacterized HTH-type transcriptional regulator RhmR
AT4157R_v1_490017	DSM301R_v1_600015	CDS	repE	Replication initiation protein, FIA
AT4157R_v1_490010	DSM301R_v1_600022	CDS		K88 minor fimbrial subunit faeH
AT4157R_v1_490016	DSM301R_v1_600016	CDS	resD	Resolvase
AT4157R_v1_490034	DSM301R_v1_1390044	CDS	yafZ	conserved hypothetical protein; CP4-6 prophage
AT4157R_v1_970029	DSM301R_v1_350006	CDS	cpdA	3',5'-cyclic adenosine monophosphate phosphodiesterase CpdA
AT4157R_v1_310002	DSM301R_v1_180012	CDS	yfeB	Chelated iron transport system membrane protein YfeB
AT4157R_v1_490024	DSM301R_v1_600008	CDS		conserved protein of unknown function
AT4157R_v1_970003	DSM301R_v1_820003	CDS	yacC	putative exoribonuclease YacC
AT4157R_v1_310003	DSM301R_v1_1390001	CDS	yfeC	Chelated iron transport system membrane protein YfeC
AT4157R_v1_310004	DSM301R_v1_180010	CDS	yfeD	Chelated iron transport system membrane protein YfeD
AT4157R_v1_490077	DSM301R_v1_180011	CDS	repA1	Replication initiation protein
AT4157R_v1_970031	DSM301R_v1_350008	CDS		conserved membrane protein of unknown function
AT4157R_v1_970030	DSM301R_v1_350007	CDS		conserved membrane protein of unknown function
AT4157R_v1_310001	DSM301R_v1_180013	CDS	hpf	putative metal ABC transporter substrate-binding protein Hpf
AT4157R_v1_490020	DSM301R_v1_600012	CDS		conserved protein of unknown function
AT4157R_v1_310010	DSM301R_v1_180004	CDS	iucB	N(6)-hydroxylysine O-acetyltransferase

AT4157R_v1_970033	DSM301R_v1_350010	CDS	kdgT	2-keto-3-deoxy-D-gluconate transporter
AT4157R_v1_490019	DSM301R_v1_600013	CDS	sopB	Protein SopB
AT4157R_v1_970042	DSM301R_v1_350019	CDS	repA	RepFIB replication protein A
AT4157R_v1_490052	DSM301R_v1_1390026	CDS	traU	Protein TraU
AT4157R_v1_490001	DSM301R_v1_600030	CDS		Helicase UvrD
AT4157R_v1_970005	DSM301R_v1_820005	CDS		transposase
AT4157R_v1_970008	DSM301R_v1_820008	CDS		Transcriptional regulator CKO_02662, LacI family
AT4157R_v1_970007	DSM301R_v1_820007	CDS		Inosine-uridine preferring nucleoside hydrolase
AT4157R_v1_970028	DSM301R_v1_350005	CDS	ugpC	sn-glycerol-3-phosphate import ATP-binding protein UgpC
AT4157R_v1_490012	DSM301R_v1_600020	CDS		conserved protein of unknown function
AT4157R_v1_970010	DSM301R_v1_820010	fCDS	ydfJ	fragment of putative transporter (part 1)
AT4157R_v1_490018	DSM301R_v1_600014	CDS	sopA	Protein SopA
AT4157R_v1_970039	DSM301R_v1_350016	CDS		conserved membrane protein of unknown function
AT4157R_v1_970020	DSM301R_v1_820020	CDS		putative Aspartate transaminase
AT4157R_v1_310008	DSM301R_v1_180006	CDS	shiF	Transport protein ShiF
AT4157R_v1_310012	DSM301R_v1_180002	CDS	iucD	L-lysine N6-monooxygenase
AT4157R_v1_970027	DSM301R_v1_350004	CDS		Glycerol-3-phosphate-binding protein
AT4157R_v1_2030003	DSM301R_v1_600003	CDS		conserved protein of unknown function
AT4157R_v1_490043	DSM301R_v1_1390035	CDS	traB	Protein TraB
AT4157R_v1_970019	DSM301R_v1_820019	CDS		Uncharacterized Na(+)/H(+) antiporter HI_1107
AT4157R_v1_490002	DSM301R_v1_600029	CDS		conserved protein of unknown function
AT4157R_v1_310009	DSM301R_v1_180005	CDS	iucA	N(2)-citryl-N(6)-acetyl-N(6)-hydroxylysine synthase
AT4157R_v1_310011	DSM301R_v1_180003	CDS	iucC	Aerobactin synthase
AT4157R_v1_490055	DSM301R_v1_1390023	CDS	traN	Protein TraN
AT4157R_v1_490030	DSM301R_v1_1390048	CDS		conserved protein of unknown function
AT4157R_v1_310013	DSM301R_v1_180001	CDS	iutA	Ferric aerobactin receptor
AT4157R_v1_490069	DSM301R_v1_1390009	CDS	traD	Coupling protein TraD
AT4157R_v1_490049	DSM301R_v1_1390029	CDS	traC	Protein TraC
AT4157R_v1_490065	DSM301R_v1_1390013	CDS	traG	Protein TraG
AT4157R_v1_490005	DSM301R_v1_600026	CDS		conserved protein of unknown function
AT4157R_v1_490071	DSM301R_v1_1390007	CDS	traI	Multifunctional conjugation protein TraI [Includes: DNA relaxase TraI ; DNA helicase I]

*Genes can be retrieved on MicroScope platform using their labels
