# Supplementary material for: The Odyssey of the Ancestral Escherich Strain through Culture Collections: an Example of Allopatric Diversification
Source: mSphere. 2018 Jan 31;3(1):e00553-17. doi: 10.1128/mSphere.00553-17 (PMC5793043; doi:10.1128/mSphere.00553-17)
Supplement: TABLE S2 [file sph001182464st2.pdf]

TABLE S2. Clusters of Orthologous Groups (COG) distribution in the NCTC86_Dunne and in genes affected by mutation in the other ancestral Escherich strain isolates

COG Classes	NCTC86_Dunne	NCTC86_Meric	NCTC86	CIP61.11	ATCC4157	DSM301
	Count	Count	Count	Count	Count	Count
Amino acid transport and metabolism	459	1	3	15	58	67
Carbohydrate transport and metabolism	464	3	7	14	76	85
Cell cycle control, cell division, chromosome partitioning	41	0	0	6	9	12
Cell motility	118	1	1	8	17	20
Cell wall/membrane/envelope biogenesis	271	2	2	13	39	38
Coenzyme transport and metabolism	153	1	1	5	20	24
Defense mechanisms	56	0	0	3	8	9
Energy production and conversion	323	4	6	12	47	47
Extracellular structures	4	0	1	0	1	1
Function unknown	347	1	0	8	31	36
General function prediction only	550	6	10	17	75	85
Inorganic ion transport and metabolism	297	1	4	11	40	44
Intracellular trafficking, secretion, and vesicular transport	162	1	3	8	24	27
Lipid transport and metabolism	107	1	1	4	20	24
Nucleotide transport and metabolism	96	1	2	7	18	20
Posttranslational modification, protein turnover, chaperones	149	0	0	2	23	23
Replication, recombination and repair	349	3	4	15	38	44
RNA processing and modification	3	0	0	2	2	2
Secondary metabolites biosynthesis, transport and catabolism	92	1	1	2	12	17
Signal transduction mechanisms	220	0	0	11	45	61
Transcription	357	5	7	18	60	65
Translation, ribosomal structure and biogenesis	183	2	2	7	18	21
