# Supplementary material for: The Odyssey of the Ancestral Escherich Strain through Culture Collections: an Example of Allopatric Diversification
Source: mSphere. 2018 Jan 31;3(1):e00553-17. doi: 10.1128/mSphere.00553-17 (PMC5793043; doi:10.1128/mSphere.00553-17)
Supplement: TABLE S3 [file sph001182464st3.pdf]

TABLE S3. Mutations found in mutator genes in ancestral Escherich strain isolates and prediction of their functionnal effect

Genes	Gene products	Mutations at the protein level*					Predictions of functional effect			Frequency of the observed allele in Uniprot
		NCTC86_Meric	NCTC86	CIP61.11	ATCC4157	DSM301	SIFT	PolyPhen-2	PROVEAN	
dnaQ (mutD)	Ɛ subunit of DNA polymerase III	-	-	-	-	-				
polC (dnaE)	α subunit of DNA polymerase III	-	-	-	-	-				
polA	DNA polymerase I	-	-	-	-	-				
mutT	Nucleoside triphosphatase	Frameshift (truncated)	Frameshift (truncated)	Frameshift (truncated)	Frameshift (truncated)	Frameshift (truncated)				
dam	DNA adenine methyltransferase	P141T	P141T	P141T	P141T	P141T	0.08	Probably damaging (0.990)	Deleterious (-7.836)	0/106
mutS	DNA mismatch repair protein MutS	V785I	V785I	V785I	V785I	V785I	0.02	Possibly damaging (0.790)	Neutral (-0.818)	0/233
mutL	DNA mismatch repair protein MutL	68delL	68delL	68delL	68delL	68delL				
		69delA	69delA	69delA	69delA	69delA				
mutH	Endonuclease	-	-	-	-	-				
uvrD	DNA helicase II	E152V	E152V	E152V	E152V	E152V	0.00	Benign (0.167)	Deleterious (-5.393)	0/132
mutY	DNA glycosylase	A217S	A217S	A217S	A217S	A217S	0.70	Benign (0.000)	Neutral (-0.028)	72/104
		A280S	A280S	A280S	A280S	A280S	0.80	Benign (0.000)	Neutral (0.704)	19/104
mutM	DNA glycosylase	S98N	S98N	S98N	S98N	S98N	0.67	Benign (0.000)	Neutral (-0.459)	19/217
		T127A	T127A	T127A	T127A	T127A	1.00	Benign (0.000)	Neutral (0.577)	189/217
miaA	Transferase	-	-	-	N163H	N163H	0.08	Probably damaging (1.000)	Deleterious (-4.47)	0/216
sodA	Superoxide dismutases	-	-	-	-	-				
sodB	Superoxide dismutases	-	-	-	V71A	V71A	0.46	Possibly damaging (0.899)	Deleterious (-3.522)	0/71
oxyR	Regulatory protein	-	-	-	-	-				
nth	Glycosylase and abasic-lyase activity	-	-	-	-	-				
nei	Glycosylase and abasic-lyase activity	-	-	-	-	-				
xthA	Nuclease	-	-	-	-	-				
nfo	Nuclease	-	-	-	-	-				
ung	Uracil glycosylase	-	-	-	-	-				
vsr	Endonuclease	A2V	A2V	A2V	A2V	A2V	0.26	Possibly damaging (0.562)	Neutral (-1.537)	17/95
		S45D	S45D	S45D	S45D	S45D	0.75	Benign (0.000)	Neutral (1.067)	3/95
		E128A	E128A	E128A	E128A	E128A	0.00 (low confidence)	Benign (0.000)	Neutral (-0.534)	23/95
ada	Methyltransferase	-	-	-	-	-				
ogt	Methyltransferase	-	-	-	-	-				
recA	DNA-binding protein	-	-	-	-	-				
recG	DNA helicase	A8T	A8T	A8T	A8T	A8T	0.00 (low confidence)	Benign (0.000)	Neutral (-0.127)	19/247
		A109T	A109T	A109T	A109T	A109T	0.33	Benign (0.000)	Neutral (-0.645)	210/247
		R282H	R282H	R282H	R282H	R282H	0.07	Benign (0.000)	Neutral (-0.027)	36/247
		E347K	E347K	E347K	E347K	E347K	0.75	Benign (0.153)	Neutral (-0.225)	15/247
		A362S	A362S	A362S	A362S	A362S	0.31	Benign (0.113)	Neutral (-0.407)	63/247
		H476R	H476R	H476R	H476R	H476R	0.54	Benign (0.000)	Neutral (2.402)	222/247
hns	DNA-binding protein	-	-	-	-	-				
topB (mutR)	DNA topoisomerase III	-	-	L474V	-	-	0.54	Benign (0.000)	Neutral (-0.392)	1/254
ssb	Single-stranded DNA-binding protein	-	-	-	-	-				

*Mutations predicted to be deleterious at the protein level at least with two of the three softwares and absent in Uniprot database are highlighted in red
