# Supplementary material for: The Odyssey of the Ancestral Escherich Strain through Culture Collections: an Example of Allopatric Diversification
Source: mSphere. 2018 Jan 31;3(1):e00553-17. doi: 10.1128/mSphere.00553-17 (PMC5793043; doi:10.1128/mSphere.00553-17)
Supplement: TABLE S4 [file sph001182464st4.pdf]

TABLE S4. Minimum Inhibitory Concentration (mg/L) values of antibiotics for the *a* ncestral Escherich strain isolates

Antibiotics	NCTC86	CIP61.11	ATCC4157	DSM301	Breakpoints*	HS(<=1%)[£]	VS(<=5%)[£]
benzylpenicillin	2 (sq)[$]	1.5 (sq)[$]	0.5 (sq)[$]	0.25 (sq)[$]	NC[§]	-	-
amoxicillin	1	0.5	0.75	0.75	8	0.5	1
ampicillin	0.5	0.5	0.19 (sq)[$]	0.19	8	0.5	ND[€]
piperacillin	0.125 (sq)[$]	0.064	0.064	0.064 (sq)[$]	8	0.25	0.5
amoxicillin / clavulanic acid	1	0.5	0.75	0.75	8	ND[€]	ND[€]
ticarcillin / clavulanic acid	0.5	0.38	0.38	0.38	8	0.5	ND[€]
cefalotin	1 (sq)[$]	0.75	2 (sq)[$]	1 (sq)[$]	32[£]	1	2
cefoxitin	1 (sq)[$]	0.75	0.75	0.75	8	0.5	ND[€]
cefixim	0.032 (sq)[$]	<0.016	<0.016 (sq)[$]	<0.016 (sq)[$]	1	0.032	ND[€]
cefotaxim	0.006	<0.002	<0.002 (sq)[$]	< 0.002	1	0.008	0.016
ceftazidim	0.023	0.032	0.023	0.023	1	0.016	0.032
aztreonam	<0.016	<0.016	<0.016	<0.016	1	0.016	ND[€]
cefepim	<0.016	<0.016	<0.016	<0.016	1	ND[€]	0.008
ceftriaxone	0.002 (sq)[$]	0.002	<0.002 (sq)[$]	<0.002 (sq)[$]	1	ND[€]	0.008
ertapenem	0.004	0.003	0.006	0.004	0.5	ND[€]	ND[€]
imipenem	0.094	0.094	0.19	0.125	2	0.016	0.032
chloramphenicol	0.5	<0.38	0.5	0.5	8	1	ND[€]
tetracyclin	0.25	0.125	0.25	0.25	8[£]	0.25	0.5
tigecyclin	0.125	0.25	0.094	0.125	1	0.032	ND[€]
colistin	0.094	0.064	0.094	0.094	2	0.064	0.125
nalidixic acid	0.5	0.25	0.25	0.25	16	0.5	ND[€]
levofloxacin	0.006 (sq)[$]	0.002 (sq)[$]	0.006 (sq)[$]	0.003 (sq)[$]	0.5	0.008	0.016
ofloxacin	0.008 (sq)[$]	0.008 (sq)[$]	0.008 (sq)[$]	0.006 (sq)[$]	0.25	0.016	ND[€]
ciprofloxacin	<0.002	<0.002	<0.002	<0.002	0.25	0.002	0.004
norfloxacin	<0.016	<0.016	0.016	<0.016	0.5	0.016	ND[€]
moxifloxacin	0.008 (sq)[$]	0.008	0.004	0.003 (sq)[$]	0.25	0.008	0.016
kanamycin	1	1	0.75	1	8[£]	0.125	0.25
amikacin	1	1	1	1.5	8	0.25	0.5
streptomycin	1.5	1	1	1.5	16[£]	1	ND[€]
gentamicin	0.125	0.125	0.125	0.125	2	0.125	ND[€]
tobramycin	0.25	0.19	0.19	0.25	2	0.125	0.25
trimethoprim	0.016	0.012	0.032	0.032	2	ND[€]	0.064
cotrimoxazol	0.008	0.008	0.012	0.016	2	0.016	ND[€]
erythromycin	64	256	48	32	NC[§]	-	-
azithromycin	2	8	2	2	16	0.5	1
fosfomycin	0.19	0.19	0.5	0.38	32	0.064	0.25

*Breakpoints are clinical breakpoints from the CASFM-EUCAST (V1.0 2017/03), or Epidemiological Cut-OFF ([£]) from EUCAST if clinical breakpoint unavailable

[£]Using the MIC distribution from EUCAST we defined hypersusceptible (HS) (green) and Very-susceptible (VS) (blue) cut-off as the lowest MIC encompassing 1 and 5% of the *E. coli* strains, respectively

[$]The presence of squatter colonies is mentionned by (sq)

[§]NC : no cut-off (clinical or epidemiological) provided by EUCAST

[€]ND : not determined
