# Supplementary material for: The Odyssey of the Ancestral Escherich Strain through Culture Collections: an Example of Allopatric Diversification
Source: mSphere. 2018 Jan 31;3(1):e00553-17. doi: 10.1128/mSphere.00553-17 (PMC5793043; doi:10.1128/mSphere.00553-17)
Supplement: TABLE S5 [file sph001182464st5.pdf]

TABLE S5. Mutations and predictions of protein change on candidate genes to hypersusceptibility of the ancestral Escherich strain isolates

Genes	Gene products	Mutations at the protein level*				Predictions of functional effect			Frequency of the observed allele in Uniprot
		NCTC86	CIP61.11	ATCC4157	DSM301	SIFT[£]	PolyPhen-2[$]	PROVEAN[§]	
acrA	Sub-unit of efflux pump AcrAB-TolC	-	-	-	-				
acrB	Sub-unit of efflux pump AcrAB-TolC	-	-	K659N	K659N	0.20	Benign (0.001)	Deleterious (-2.666)	1/79
		G679S	G679S	G679S	G679S	0.00	Probably damaging (0.998)	Deleterious (-5.837)	0/79
tolC	Sub-unit of multi druf efflux systems	-	-	-	-				
acrD	Sub-unit of efflux pump AcrAD-TolC	-	-	-	-				
acrE	Sub-unit of efflux pump AcrEF-TolC	G191D	G191D	G191D	G191D	0.00	Probably damaging (1.000)	Deleterious (-6.973)	0/46
acrR	DNA binding transcriptional repressor	-	-	-	-				
acrZ	AcrB transmembran domain associated protein	-	-	-	-				
acrS	Regulator/repressor of efflux pump AcrEF-TolC	W50R	W50R	W50R	W50R	0.00	Probably damaging (0.957)	Deleterious (-13.78)	0/42
marA	DNA binding transcriptional activator	-	-	-	-				
marB	DNA binding transcriptional activator	-	-	-	-				
marR	DNA binding transcriptional repressor	-	-	-	-				
soxS	DNA binding transcriptional regulator	-	-	-	-				
soxR	DNA binding transcriptional dual regulator	-	-	-	-				
evgS	Hybrid sensory histidine kinase in two-component regulatory system	F152L	F152L	F152L	F152L	0.02	Benign (0.006)	Neutral (0.414)	83/83
evgA	DNA binding transcriptional activator	-	-	-	-				
phoP	DNA binding transcriptional regulator	-	K149T	-	-	0.23	Benign (0.002)	Neutral (-1.112)	0/87
phoQ	Sensor protein	-	-	-	-				
sdiA	DNA binding transcriptional activator	-	-	-	-				
rob	DNA binding transcriptional activator	G5C	G5C	G5C	G5C	0.01	Possibly damaging (0.822)	Deleterious (-3.302)	0/86
yajC	Preprotein translocase subunit	-	-	-	-				
mdtC	Multidrug resistance protein	I46M	I46M	I46M	I46M	0.22	Benign (0,000)	Neutral (0.893)	241/253
		V208I	V208I	V208I	V208I	1.00	Benign (0,000)	Neutral (0.899)	222/253
		S365A	S365A	S365A	S365A	1.00	Benign (0,000)	Neutral (2.181)	184/253
		L387I	L387I	L387I	L387I	0.00	Benign (0,394)	Neutral (-1.572)	4/253
		G472V	G472V	G472V	G472V	0.18	Benign (0,000)	Neutral (3.512)	17/253
mdtB	Multidrug resistance protein	L910M	L910M	L910M	L910M	0.18	Benign (0,000)	Neutral (0.013)	152/248
emrA	Sub-unit of efflux pump EmrAB-TolC	-	-	-	-				
emrB	Sub-unit of efflux pump EmrAB-TolC	-	-	-	-				
emrY	Sub-unit of efflux pump EmrYK-TolC	-	-	-	-				
emrK	Sub-unit of efflux pump EmrYK-TolC	-	-	-	-				
ompA	Outer-membran protein A (porin)	-	-	-	-				
ompF	Outer-membran protein F (porin)	E139G	E139G	E139G	E139G	0.04	Possibly damaging (0.893)	Deleterious (-4.851)	0/101
ompC	Outer-membran porin protein C	-	-	-	-				
ompR	Osmolarity response regulator	T149A	T149A	T149A	T149A	0.28	Benign (0,001)	Deleterious (-2.503)	0/115
envZ	Osmolarity sensor protein	-	-	-	-				

*Mutations predicted to be deleterious at the protein level at least with two of the three softwares and absent in Uniprot database are highlighted in red

[£]SIFT software : a score between 0 and 0,05 predicted an alteration of the protein function (altereted) ; a score above 0,05 predicted no alteration (tolerated) : *Kumar et al* . 2009. Nature protocol. 4:1073-1081

[$]Polyphen-2 software : 3 classes of damaging were predicted : benign, possibly damaged and probably damaged described as follow : *Adzhubei et al* . 2010. Nature methods. 7(4): 248-249

[§]PROVEAN : a score < -2,5 is predicted as deleterious : Choi *et al.,* 2015.Bioinformatics 31(16): 2745-2747.
