# Supplementary material for: The Odyssey of the Ancestral Escherich Strain through Culture Collections: an Example of Allopatric Diversification
Source: mSphere. 2018 Jan 31;3(1):e00553-17. doi: 10.1128/mSphere.00553-17 (PMC5793043; doi:10.1128/mSphere.00553-17)
Supplement: TABLE S6 [file sph001182464st6.pdf]

TABLE S6. List of genes exhibiting Indels and non-synonymous or non-sense SNPs in squatter colonies of the ancestral Escherich strain isolates and absent in the parental isolate

Squatter colonies isolates	Label of NCTC86_Dunne gene	Gene description	Mutation type	Effect
ATCC4157_S1	NCTC86EC_RS02620	2-succinyl-5-enolpyruvyl-6-hydroxy-3- cyclohexene-1-carboxylate synthase	Indel	
ATCC4157_S1	NCTC86EC_RS04290	non-ribosomal peptide synthetase	SNP	nonsyn
ATCC4157_S1	NCTC86EC_RS05595	hypothetical protein	SNP	nonsyn
ATCC4157_S1	NCTC86EC_RS06855	hypothetical protein	SNP	nonsyn
ATCC4157_S1	NCTC86EC_RS08170	rha family phage regulatory protein	SNP	nonsyn
ATCC4157_S1	NCTC86EC_RS10725	malate transporter	Indel	
ATCC4157_S1	NCTC86EC_RS10750	hypothetical protein	Indel	
ATCC4157_S1	NCTC86EC_RS12600	IS4 family transposase	Indel	
ATCC4157_S1	NCTC86EC_RS13085	ureidoglycolate dehydrogenase (NAD(+))	SNP	nonsyn
ATCC4157_S1	NCTC86EC_RS14715	RHS element protein	Indel	
ATCC4157_S1	NCTC86EC_RS16645	glutathione synthetase	SNP	nonsyn
ATCC4157_S1	NCTC86EC_RS19370	two-component system response regulator OmpR	SNP	nonsyn
ATCC4157_S1	NCTC86EC_RS20360	xylulokinase	Indel	
ATCC4157_S1	NCTC86EC_RS20525	hypothetical protein	Indel	
ATCC4157_S1	NCTC86EC_RS22675	stress-induced protein	SNP	nonsyn
ATCC4157_S1	NCTC86EC_RS23355	antitoxin	SNP	nonsyn
ATCC4157_S1	NCTC86EC_RS23370	hypothetical protein	SNP	nonsyn
ATCC4157_S1	NCTC86EC_RS23370	hypothetical protein	SNP	nonsyn
ATCC4157_S1	NCTC86EC_RS23370	hypothetical protein	SNP	nonsyn
ATCC4157_S1	NCTC86EC_RS23370	hypothetical protein	SNP	nonsyn
ATCC4157_S1	NCTC86EC_RS23535	glycyl radical enzyme	SNP	nonsyn
ATCC4157_S1	NCTC86EC_RS24775	hypothetical protein	SNP	nonsyn
ATCC4157_S1	NCTC86EC_RS24785	GTPase	SNP	nonsyn
ATCC4157_S2	NCTC86EC_RS00295	molecular chaperone DjlA	SNP	nonsyn
ATCC4157_S2	NCTC86EC_RS00585	transcriptional regulator PdhR	SNP	nonsyn
ATCC4157_S2	NCTC86EC_RS00795	glutamate-1-semialdehyde 2,1-aminomutase	SNP	nonsyn
ATCC4157_S2	NCTC86EC_RS01095	uracil-DNA glycosylase	SNP	nonsense
ATCC4157_S2	NCTC86EC_RS01345	stationary phase inducible protein CsiE	SNP	nonsyn
ATCC4157_S2	NCTC86EC_RS01365	Fe-S cluster assembly transcriptional regulator IscR	SNP	nonsyn
ATCC4157_S2	NCTC86EC_RS01660	tRNA cytosine(34) acetyltransferase TmcA	SNP	nonsyn
ATCC4157_S2	NCTC86EC_RS01785	eutA ethanolamine utilization protein EutA	SNP	nonsense
ATCC4157_S2	NCTC86EC_RS03120	multiphosphoryl transfer protein	SNP	nonsyn
ATCC4157_S2	NCTC86EC_RS03640	diguanylate cyclase	SNP	nonsyn
ATCC4157_S2	NCTC86EC_RS04060	hypothetical protein	SNP	nonsyn
ATCC4157_S2	NCTC86EC_RS04285	polyketide synthase	Indel	
ATCC4157_S2	NCTC86EC_RS04310	MFS transporter	SNP	nonsyn
ATCC4157_S2	NCTC86EC_RS04370	sensor histidine kinase	SNP	nonsyn
ATCC4157_S2	NCTC86EC_RS04510	flagellar motor switch protein FliG	SNP	nonsyn
ATCC4157_S2	NCTC86EC_RS05820	hypothetical protein	SNP	nonsyn
ATCC4157_S2	NCTC86EC_RS05905	4Fe-4S ferredoxin	SNP	nonsyn
ATCC4157_S2	NCTC86EC_RS06250	arginine/ornithine antiporter transporter	SNP	nonsyn
ATCC4157_S2	NCTC86EC_RS06365	dimethyl sulfoxide reductase subunit A	SNP	nonsyn
ATCC4157_S2	NCTC86EC_RS06560	autoinducer 2 ABC transporter substrate-binding protein	Indel	
ATCC4157_S2	NCTC86EC_RS06580	LsrR family transcriptional regulator	SNP	nonsyn
ATCC4157_S2	NCTC86EC_RS06590	hypothetical protein	Indel	
ATCC4157_S2	NCTC86EC_RS06725	peptide ABC transporter ATP-binding protein	Indel	
ATCC4157_S2	NCTC86EC_RS06855	hypothetical protein	SNP	nonsyn
ATCC4157_S2	NCTC86EC_RS07095	hypothetical protein	SNP	nonsyn
ATCC4157_S2	NCTC86EC_RS07110	hypothetical protein	SNP	nonsyn
ATCC4157_S2	NCTC86EC_RS07205	1,2-phenylacetyl-CoA epoxidase subunit A	SNP	nonsyn
ATCC4157_S2	NCTC86EC_RS07370	periplasmic murein peptide-binding protein	SNP	nonsyn
ATCC4157_S2	NCTC86EC_RS07680	LysR family transcriptional regulator CysB	SNP	nonsyn
ATCC4157_S2	NCTC86EC_RS07835	phage tail fiber protein	Indel	
ATCC4157_S2	NCTC86EC_RS08515	sigma-54-dependent Fis family transcriptional regulator	SNP	nonsyn
ATCC4157_S2	NCTC86EC_RS08565	K+/H+ antiporter NhaP2	SNP	nonsyn
ATCC4157_S2	NCTC86EC_RS08990	transcription-repair coupling factor	SNP	nonsyn
ATCC4157_S2	NCTC86EC_RS10115	fimbrial-like adhesin protein	Indel	
ATCC4157_S2	NCTC86EC_RS10480	hypothetical protein	SNP	nonsyn
ATCC4157_S2	NCTC86EC_RS10665	IS66 family transposase	SNP	nonsyn
ATCC4157_S2	NCTC86EC_RS10725	malate transporter	Indel	
ATCC4157_S2	NCTC86EC_RS10750	hypothetical protein	Indel	
ATCC4157_S2	NCTC86EC_RS10990	nuclease	Indel	
ATCC4157_S2	NCTC86EC_RS11255	multidrug transporter MdfA	SNP	nonsyn
ATCC4157_S2	NCTC86EC_RS11350	glycyl radical enzyme	SNP	nonsyn
ATCC4157_S2	NCTC86EC_RS11380	transcriptional regulator MntR	SNP	nonsyn
ATCC4157_S2	NCTC86EC_RS11860	succinyl-CoA ligase subunit beta	SNP	nonsyn
ATCC4157_S2	NCTC86EC_RS11870	2-oxoglutarate dehydrogenase subunit E1	SNP	nonsyn
ATCC4157_S2	NCTC86EC_RS12405	dpiB sensor histidine kinase	SNP	nonsyn
ATCC4157_S2	NCTC86EC_RS12600	IS4 family transposase	Indel	
ATCC4157_S2	NCTC86EC_RS12605	protein HokE	SNP	nonsyn
ATCC4157_S2	NCTC86EC_RS12635	miniconductance mechanosensitive channel YbdG	SNP	nonsyn
ATCC4157_S2	NCTC86EC_RS12670	two-component sensor histidine kinase	SNP	nonsyn
ATCC4157_S2	NCTC86EC_RS12715	methyltransferase	SNP	nonsyn
ATCC4157_S2	NCTC86EC_RS13285	ferrochelatase	SNP	nonsyn
ATCC4157_S2	NCTC86EC_RS13350	MexE family multidrug efflux RND transporter periplasmic adaptor subunit	SNP	nonsyn
ATCC4157_S2	NCTC86EC_RS14295	PaoABC aldehyde oxidoreductase Moco-containing subunit	SNP	nonsyn
ATCC4157_S2	NCTC86ECAM_2901	transposase	Indel	
ATCC4157_S2	NCTC86EC_RS14770	type VI secretion system protein ImpI	SNP	nonsyn
ATCC4157_S2	NCTC86EC_RS15720	rumA 23S rRNA (uracil(1939)-C(5))-methyltransferase RlmD	SNP	nonsyn
ATCC4157_S2	NCTC86EC_RS15730	glucarate dehydratase	SNP	nonsyn
ATCC4157_S2	NCTC86EC_RS15905	pitrilysin	SNP	nonsyn
ATCC4157_S2	NCTC86EC_RS15990	bifunctional 2-acylglycerophosphoethanolamine acyltransferase/acyl-ACP synthetase	SNP	nonsyn
ATCC4157_S2	NCTC86EC_RS16165	type III secretion system protein	SNP	nonsyn
ATCC4157_S2	NCTC86EC_RS16215	sigma-54-dependent Fis family transcriptional regulator	SNP	nonsyn
ATCC4157_S2	NCTC86EC_RS16380	6-phospho-beta-glucosidase	SNP	nonsyn
ATCC4157_S2	NCTC86EC_RS16620	S-adenosylmethionine synthase	SNP	nonsyn
ATCC4157_S2	NCTC86EC_RS16770	hypothetical protein	SNP	nonsyn
ATCC4157_S2	NCTC86EC_RS17420	two-component sensor histidine kinase	SNP	nonsyn
ATCC4157_S2	NCTC86EC_RS17460	ADP-ribose pyrophosphatase	SNP	nonsyn
ATCC4157_S2	NCTC86EC_RS17495	DUF4051 domain-containing protein	SNP	nonsyn
ATCC4157_S2	NCTC86EC_RS17615	transcriptional activator TtdR	SNP	nonsyn
ATCC4157_S2	NCTC86EC_RS18035	outer membrane usher protein YraJ	SNP	nonsyn
ATCC4157_S2	NCTC86EC_RS18065	osmotically-inducible protein OsmY	SNP	nonsyn
ATCC4157_S2	NCTC86EC_RS18070	permease	Indel	
ATCC4157_S2	NCTC86EC_RS18160	translation initiation factor IF-2	SNP	nonsyn
ATCC4157_S2	NCTC86EC_RS18270	acid stress protein IbaG	SNP	nonsyn
ATCC4157_S2	NCTC86EC_RS18300	hypothetical protein	SNP	nonsyn
ATCC4157_S2	NCTC86EC_RS18400	outer membrane fimbrial subunit usher protein	SNP	nonsyn
ATCC4157_S2	NCTC86EC_RS18510	IS4 family transposase	SNP	nonsyn
ATCC4157_S2	NCTC86EC_RS18550	ribonuclease E/G	SNP	nonsyn
ATCC4157_S2	NCTC86EC_RS19195	frlA fructoselysine transporter	SNP	nonsyn
ATCC4157_S2	NCTC86EC_RS19370	two-component system response regulator OmpR	SNP	nonsyn
ATCC4157_S2	NCTC86EC_RS19380	transcription accessory protein	SNP	nonsyn
ATCC4157_S2	NCTC86EC_RS19760	ISAs1 family transposase	SNP	nonsyn

ATCC4157_S2	NCTC86EC_RS20515	RHS element protein	SNP	nonsyn
ATCC4157_S2	NCTC86EC_RS20525	hypothetical protein	Indel	
ATCC4157_S2	NCTC86EC_RS21315	trkD low affinity potassium transport system protein kup	Indel	
ATCC4157_S2	NCTC86EC_RS21435	transcriptional regulator	SNP	nonsyn
ATCC4157_S2	NCTC86EC_RS21975	aldose 1-epimerase	SNP	nonsyn
ATCC4157_S2	NCTC86EC_RS22010	membrane protein	SNP	nonsyn
ATCC4157_S2	NCTC86EC_RS22230	aquaporin	SNP	nonsyn
ATCC4157_S2	NCTC86EC_RS22380	phosphoenolpyruvate carboxylase	SNP	nonsyn
ATCC4157_S2	NCTC86EC_RS22395	N-acetyl-gamma-glutamyl-phosphate reductase	SNP	nonsyn
ATCC4157_S2	NCTC86EC_RS22845	glycerol-3-phosphate 1-O-acyltransferase	SNP	nonsyn
ATCC4157_S2	NCTC86EC_RS22970	Na+/H+ antiporter	SNP	nonsyn
ATCC4157_S2	NCTC86EC_RS23030	glutamate/aspartate:proton symporter GltP	SNP	nonsyn
ATCC4157_S2	NCTC86EC_RS23050	multidrug resistance outer membrane protein MdtP	SNP	nonsyn
ATCC4157_S2	NCTC86EC_RS23200	YjcZ family protein; yhjH motility defect suppressor	SNP	nonsyn
ATCC4157_S2	NCTC86EC_RS23330	cadB cadaverine/lysine antiporter	SNP	nonsyn
ATCC4157_S2	NCTC86EC_RS23355	antitoxin	SNP	nonsyn
ATCC4157_S2	NCTC86EC_RS23370	hypothetical protein	SNP	nonsyn
ATCC4157_S2	NCTC86EC_RS23370	hypothetical protein	SNP	nonsyn
ATCC4157_S2	NCTC86EC_RS23370	hypothetical protein	SNP	nonsyn
ATCC4157_S2	NCTC86EC_RS23370	hypothetical protein	SNP	nonsyn
ATCC4157_S2	NCTC86EC_RS23440	pseudouridine kinase	SNP	nonsyn
ATCC4157_S2	NCTC86EC_RS23450	nucleoside permease	SNP	nonsyn
ATCC4157_S2	NCTC86EC_RS23750	hypothetical protein	SNP	nonsyn
ATCC4157_S2	NCTC86EC_RS23760	molecular chaperone GroEL	SNP	nonsyn
ATCC4157_S2	NCTC86EC_RS24115	OapA family protein	SNP	nonsyn
ATCC4157_S2	NCTC86EC_RS24775	hypothetical protein	SNP	nonsyn
ATCC4157_S2	NCTC86EC_RS24785	GTPase	SNP	nonsyn
ATCC4157_S2	NCTC86EC_RS25005	isoaspartyl dipeptidase	SNP	nonsyn
ATCC4157_S2	NCTC86EC_RS25280	lplA lipoate--protein ligase	SNP	nonsyn
ATCC4157_S3	NCTC86EC_RS01660	tRNA cytosine(34) acetyltransferase TmcA	SNP	nonsyn
ATCC4157_S3	NCTC86EC_RS03640	diguanylate cyclase	SNP	nonsyn
ATCC4157_S3	NCTC86EC_RS25625	transposase	SNP	nonsyn
ATCC4157_S3	NCTC86EC_RS04290	non-ribosomal peptide synthetase	SNP	nonsyn
ATCC4157_S3	NCTC86EC_RS05740	phosphoenolpyruvate synthase	SNP	nonsyn
ATCC4157_S3	NCTC86EC_RS05820	hypothetical protein	SNP	nonsyn
ATCC4157_S3	NCTC86EC_RS05905	4Fe-4S ferredoxin	SNP	nonsyn
ATCC4157_S3	NCTC86EC_RS06590	hypothetical protein	Indel	
ATCC4157_S3	NCTC86EC_RS06855	hypothetical protein	SNP	nonsyn
ATCC4157_S3	NCTC86EC_RS08170	rha family phage regulatory protein	SNP	nonsyn
ATCC4157_S3	NCTC86EC_RS09560	transposase	SNP	nonsyn
ATCC4157_S3	NCTC86EC_RS10665	IS66 family transposase	SNP	nonsyn
ATCC4157_S3	NCTC86EC_RS10725	malate transporter	Indel	
ATCC4157_S3	NCTC86EC_RS10750	hypothetical protein	Indel	
ATCC4157_S3	NCTC86EC_RS12600	IS4 family transposase	Indel	
ATCC4157_S3	NCTC86EC_RS13350	MexE family multidrug efflux RND transporter periplasmic adaptor subunit	SNP	nonsyn
ATCC4157_S3	NCTC86EC_RS14715	RHS element protein	Indel	
ATCC4157_S3	NCTC86EC_RS16645	glutathione synthetase	SNP	nonsyn
ATCC4157_S3	NCTC86EC_RS17460	ADP-ribose pyrophosphatase	SNP	nonsyn
ATCC4157_S3	NCTC86EC_RS17615	transcriptional activator TtdR	SNP	nonsyn
ATCC4157_S3	NCTC86EC_RS17845	LysR family transcriptional regulator	SNP	nonsyn
ATCC4157_S3	NCTC86EC_RS18400	outer membrane fimbrial subunit usher protein	SNP	nonsyn
ATCC4157_S3	NCTC86EC_RS18510	IS4 family transposase	SNP	nonsyn
ATCC4157_S3	NCTC86EC_RS19370	two-component system response regulator OmpR	SNP	nonsyn
ATCC4157_S3	NCTC86EC_RS20515	RHS element protein	SNP	nonsyn
ATCC4157_S3	NCTC86EC_RS20525	hypothetical protein	Indel	
ATCC4157_S3	NCTC86EC_RS21920	two-component system sensor histidine kinase NtrB	SNP	nonsyn
ATCC4157_S3	NCTC86EC_RS22675	stress-induced protein	SNP	nonsyn
ATCC4157_S3	NCTC86EC_RS23030	glutamate/aspartate:proton symporter GltP	SNP	nonsyn
ATCC4157_S3	NCTC86EC_RS23355	antitoxin	SNP	nonsyn
ATCC4157_S3	NCTC86EC_RS23370	hypothetical protein	SNP	nonsyn
ATCC4157_S3	NCTC86EC_RS23370	hypothetical protein	SNP	nonsyn
ATCC4157_S3	NCTC86EC_RS23370	hypothetical protein	SNP	nonsyn
ATCC4157_S3	NCTC86EC_RS23370	hypothetical protein	SNP	nonsyn
ATCC4157_S3	NCTC86EC_RS23440	pseudouridine kinase	SNP	nonsyn
ATCC4157_S3	NCTC86EC_RS23450	nucleoside permease	SNP	nonsyn
ATCC4157_S3	NCTC86EC_RS23535	glycyl radical enzyme	SNP	nonsyn
ATCC4157_S3	NCTC86EC_RS23750	hypothetical protein	SNP	nonsyn
ATCC4157_S3	NCTC86EC_RS24775	hypothetical protein	SNP	nonsyn
ATCC4157_S3	NCTC86EC_RS24785	GTPase	SNP	nonsyn
NCTC86_S1	NCTC86EC_RS00135	isoleucine--tRNA ligase	SNP	nonsyn
NCTC86_S1	NCTC86EC_RS00355	thiamine-binding periplasmic protein	SNP	nonsyn
NCTC86_S1	NCTC86EC_RS00585	transcriptional regulator PdhR	SNP	nonsyn
NCTC86_S1	NCTC86EC_RS00595	aceF dihydrolipoyllysine-residue acetyltransferase component of pyruvate dehydrogenase complex	SNP	nonsyn
NCTC86_S1	NCTC86EC_RS00760	ATP-dependent helicase HrpB	Indel	
NCTC86_S1	NCTC86EC_RS01125	tRNA (adenosine(37)-N6)-methyltransferase TrmM	Indel	
NCTC86_S1	NCTC86EC_RS01160	elongation factor 4	SNP	nonsyn
NCTC86_S1	NCTC86EC_RS01275	DUF4380 domain-containing protein	SNP	nonsense
NCTC86_S1	NCTC86EC_RS01275	DUF4380 domain-containing protein	Indel	
NCTC86_S1	NCTC86EC_RS01390	Fe-S protein assembly chaperone HscA	SNP	nonsyn
NCTC86_S1	NCTC86EC_RS01600	hyfE hydrogenase-4 component E	SNP	nonsyn
NCTC86_S1	NCTC86EC_RS01640	outer membrane protein assembly factor BamC	SNP	nonsyn
NCTC86_S1	NCTC86EC_RS01890	cysteine synthase B	SNP	nonsyn
NCTC86_S1	NCTC86EC_RS02365	protein DedD	SNP	nonsyn
NCTC86_S1	NCTC86EC_RS02650	4-amino-4-deoxy-L-arabinose-phospho-UDP flippase	SNP	nonsyn
NCTC86_S1	NCTC86EC_RS02790	autotransporter outer membrane beta-barrel domain-containing protein	SNP	nonsyn
NCTC86_S1	NCTC86EC_RS02815	hypothetical protein	SNP	nonsyn
NCTC86_S1	NCTC86EC_RS02940	ferredoxin-type protein NapG	SNP	nonsyn
NCTC86_S1	NCTC86EC_RS03230	galactose/methyl galactoside import ATP-binding protein MglA	SNP	nonsyn
NCTC86_S1	NCTC86EC_RS03285	multidrug transporter permease	SNP	nonsyn
NCTC86_S1	NCTC86EC_RS03320	ABC transporter substrate-binding protein	SNP	nonsyn
NCTC86_S1	NCTC86EC_RS03490	sugar kinase	SNP	nonsyn
NCTC86_S1	NCTC86EC_RS03705	putative colanic acid polymerase WcaD	SNP	nonsyn
NCTC86_S1	NCTC86EC_RS03810	capsular biosynthesis protein	SNP	nonsyn
NCTC86_S1	NCTC86EC_RS25625	transposase	Indel	
NCTC86_S1	NCTC86EC_RS04575	RNA polymerase sigma factor FliA	SNP	nonsyn
NCTC86_S1	NCTC86EC_RS04655	tyrosine transporter TyrP	SNP	nonsyn
NCTC86_S1	NCTC86EC_RS05635	membrane protein	SNP	nonsyn
NCTC86_S1	NCTC86EC_RS06680	glutamate decarboxylase	SNP	nonsyn
NCTC86_S1	NCTC86EC_RS06805	respiratory nitrate reductase 2 alpha chain	SNP	nonsyn
NCTC86_S1	NCTC86EC_RS07275	tRNA 2-thiocytidine(32) synthetase TtcA	Indel	
NCTC86_S1	NCTC86EC_RS07275	tRNA 2-thiocytidine(32) synthetase TtcA	SNP	nonsyn
NCTC86_S1	NCTC86EC_RS07505	phage shock protein PspA	SNP	nonsyn
NCTC86_S1	NCTC86EC_RS07655	GTP cyclohydrolase II	SNP	nonsyn
NCTC86_S1	NCTC86EC_RS08270	oligopeptide ABC transporter substrate-binding protein OppA	SNP	nonsyn
NCTC86_S1	NCTC86EC_RS08510	adhesin	SNP	nonsyn
NCTC86_S1	NCTC86EC_RS09055	PTS glucose EIICB component	SNP	nonsyn
NCTC86_S1	NCTC86EC_RS09160	flgI flagellar P-ring protein	SNP	nonsyn

NCTC86_S1	NCTC86EC_RS09880	hypothetical protein	SNP	nonsyn
NCTC86_S1	NCTC86EC_RS10015	hypothetical protein	SNP	nonsyn
NCTC86_S1	NCTC86EC_RS10095	dihydroorotate dehydrogenase 2	SNP	nonsyn
NCTC86_S1	NCTC86EC_RS10270	30S ribosomal protein S1	SNP	nonsyn
NCTC86_S1	NCTC86EC_RS10315	formate C-acetyltransferase	SNP	nonsyn
NCTC86_S1	NCTC86EC_RS10360	dimethyl sulfoxide reductase subunit A	SNP	nonsyn
NCTC86_S1	NCTC86EC_RS10530	restriction endonuclease	SNP	nonsense
NCTC86_S1	NCTC86EC_RS10595	pilin outer membrane usher protein SafC	Indel	
NCTC86_S1	NCTC86EC_RS10725	malate transporter	SNP	nonsyn
NCTC86_S1	NCTC86EC_RS10745	macrolide ABC transporter ATP-binding protein	Indel	
NCTC86_S1	NCTC86EC_RS10750	hypothetical protein	Indel	
NCTC86_S1	NCTC86EC_RS10755	hypothetical protein	Indel	
NCTC86_S1	NCTC86EC_RS10840	DNA-binding protein	SNP	nonsyn
NCTC86_S1	NCTC86EC_RS11050	ATP-dependent Clp protease adaptor ClpS	SNP	nonsyn
NCTC86_S1	NCTC86EC_RS11085	aquaporin	SNP	nonsyn
NCTC86_S1	NCTC86EC_RS11095	hydroxylamine reductase	SNP	nonsyn
NCTC86_S1	NCTC86EC_RS11350	glycyl radical enzyme	SNP	nonsyn
NCTC86_S1	NCTC86EC_RS12035	two-component system sensor histidine kinase KdbD	SNP	nonsyn
NCTC86_S1	NCTC86EC_RS12130	PTS N-acetylglucosamine EIICBA component	SNP	nonsyn
NCTC86_S1	NCTC86EC_RS12495	hypothetical protein	Indel	
NCTC86_S1	NCTC86EC_RS12600	IS4 family transposase	Indel	
NCTC86_S1	NCTC86EC_RS12645	cation transporter	SNP	nonsyn
NCTC86_S1	NCTC86EC_RS12660	cation efflux system protein CusC	SNP	nonsyn
NCTC86_S1	NCTC86EC_RS13860	microcin transporter	SNP	nonsyn
NCTC86_S1	NCTC86EC_RS14015	cyanate transporter	SNP	nonsense
NCTC86_S1	NCTC86EC_RS14035	cytosine deaminase	SNP	nonsyn
NCTC86_S1	NCTC86EC_RS14050	2-methylcitrate dehydratase	SNP	nonsyn
NCTC86_S1	NCTC86EC_RS14065	propionate catabolism operon regulatory protein PrpR	SNP	nonsyn
NCTC86_S1	NCTC86EC_RS14710	hypothetical protein	Indel	
NCTC86_S1	NCTC86EC_RS14795	type VI secretion system-associated protein	SNP	nonsyn
NCTC86_S1	NCTC86EC_RS14915	chaperone protein ClpB	SNP	nonsyn
NCTC86_S1	NCTC86EC_RS15000	30S ribosomal protein S16	SNP	nonsyn
NCTC86_S1	NCTC86EC_RS15600	CRISPR-associated helicase/endonuclease Cas3	SNP	nonsyn
NCTC86_S1	NCTC86EC_RS15990	bifunctional 2-acylglycerophosphoethanolamine acyltransferase/acyl-ACP synthetase	SNP	nonsyn
NCTC86_S1	NCTC86EC_RS16450	transcriptional regulator ArgP	SNP	nonsyn
NCTC86_S1	NCTC86EC_RS16585	hypothetical protein	SNP	nonsyn
NCTC86_S1	NCTC86EC_RS17020	restriction endonuclease subunit M	SNP	nonsyn
NCTC86_S1	NCTC86EC_RS17100	polysialic acid transporter	Indel	
NCTC86_S1	NCTC86EC_RS17170	acyl-CoA synthetase	SNP	nonsyn
NCTC86_S1	NCTC86EC_RS17690	transcriptional regulator	SNP	nonsyn
NCTC86_S1	NCTC86EC_RS18655	efflux transporter periplasmic adaptor subunit	SNP	nonsyn
NCTC86_S1	NCTC86EC_RS18995	general secretory pathway protein	SNP	nonsyn
NCTC86_S1	NCTC86EC_RS19090	glutathione-regulated potassium-efflux system protein KefB	SNP	nonsyn
NCTC86_S1	NCTC86EC_RS19165	MFS transporter	SNP	nonsyn
NCTC86_S1	NCTC86EC_RS19370	two-component system response regulator OmpR	SNP	nonsyn
NCTC86_S1	NCTC86EC_RS19415	Fe-S biogenesis protein NfuA	SNP	nonsyn
NCTC86_S1	NCTC86EC_RS19435	transcriptional regulator MalT	SNP	nonsyn
NCTC86_S1	NCTC86EC_RS20515	RHS element protein	SNP	nonsyn
NCTC86_S1	NCTC86EC_RS20525	hypothetical protein	Indel	
NCTC86_S1	NCTC86EC_RS20965	sulfatase/phosphatase superfamily protein	SNP	nonsyn
NCTC86_S1	NCTC86EC_RS21495	polysaccharide chain length modulation protein	Indel	
NCTC86_S1	NCTC86EC_RS21515	glucose-1-phosphate thymidylyltransferase 2	SNP	nonsyn
NCTC86_S1	NCTC86EC_RS21540	enterobacterial common antigen polymerase	SNP	nonsyn
NCTC86_S1	NCTC86EC_RS21550	transporter	Indel	
NCTC86_S1	NCTC86EC_RS21590	uroporphyrinogen-III C-methyltransferase	SNP	nonsyn
NCTC86_S1	NCTC86EC_RS22790	malG maltose ABC transporter permease	SNP	nonsyn
NCTC86_S1	NCTC86EC_RS22830	hypothetical protein	SNP	nonsyn
NCTC86_S1	NCTC86EC_RS22975	hypothetical protein	SNP	nonsyn
NCTC86_S1	NCTC86EC_RS23145	alpha-D-ribose 1-methylphosphonate 5-phosphate C-P-lyase	SNP	nonsyn
NCTC86_S1	NCTC86EC_RS23240	arginine decarboxylase	SNP	nonsyn
NCTC86_S1	NCTC86EC_RS23450	nucleoside permease	Indel	
NCTC86_S1	NCTC86EC_RS24510	PTS glucose transporter subunit IIB	SNP	nonsyn
NCTC86_S1	NCTC86EC_RS24705	fec operon regulator FecR	SNP	nonsyn
NCTC86_S1	NCTC86EC_RS24780	hypothetical protein	Indel	
NCTC86_S1	NCTC86EC_RS24805	hypothetical protein	SNP	nonsyn
NCTC86_S1	NCTC86EC_RS24860	hypothetical protein	SNP	nonsyn
NCTC86_S1	NCTC86EC_RS25300	trifunctional nicotinamide-nucleotide adenylyltransferase/ribosylnicotinamide kinase/transcriptional regulator NadR	Indel	
NCTC86_S2	NCTC86EC_RS00135	isoleucine--tRNA ligase	SNP	nonsyn
NCTC86_S2	NCTC86EC_RS00355	thiamine-binding periplasmic protein	SNP	nonsyn
NCTC86_S2	NCTC86EC_RS00585	transcriptional regulator PdhR	SNP	nonsyn
NCTC86_S2	NCTC86EC_RS00595	aceF dihydrolipoyllysine-residue acetyltransferase component of pyruvate dehydrogenase complex	SNP	nonsyn
NCTC86_S2	NCTC86EC_RS00760	ATP-dependent helicase HrpB	Indel	
NCTC86_S2	NCTC86EC_RS01010	DL-methionine transporter substrate-binding subunit	Indel	
NCTC86_S2	NCTC86EC_RS01125	tRNA (adenosine(37)-N6)-methyltransferase TrmM	Indel	
NCTC86_S2	NCTC86EC_RS01160	elongation factor 4	SNP	nonsyn
NCTC86_S2	NCTC86EC_RS01275	DUF4380 domain-containing protein	SNP	nonsense
NCTC86_S2	NCTC86EC_RS01390	Fe-S protein assembly chaperone HscA	SNP	nonsyn
NCTC86_S2	NCTC86EC_RS01600	hyfE hydrogenase-4 component E	SNP	nonsyn
NCTC86_S2	NCTC86EC_RS01640	outer membrane protein assembly factor BamC	SNP	nonsyn
NCTC86_S2	NCTC86EC_RS01890	cysteine synthase B	SNP	nonsyn
NCTC86_S2	NCTC86EC_RS02150	transporter	SNP	nonsyn
NCTC86_S2	NCTC86EC_RS02365	protein DedD	SNP	nonsyn
NCTC86_S2	NCTC86EC_RS02650	4-amino-4-deoxy-L-arabinose-phospho-UDP flippase	SNP	nonsyn
NCTC86_S2	NCTC86EC_RS02790	autotransporter outer membrane beta-barrel domain-containing protein	SNP	nonsyn
NCTC86_S2	NCTC86EC_RS02815	hypothetical protein	SNP	nonsyn
NCTC86_S2	NCTC86EC_RS02940	ferredoxin-type protein NapG	SNP	nonsyn
NCTC86_S2	NCTC86EC_RS03230	galactose/methyl galactoside import ATP-binding protein MglA	SNP	nonsyn
NCTC86_S2	NCTC86EC_RS03285	multidrug transporter permease	SNP	nonsyn
NCTC86_S2	NCTC86EC_RS03320	ABC transporter substrate-binding protein	SNP	nonsyn
NCTC86_S2	NCTC86EC_RS03490	sugar kinase	SNP	nonsyn
NCTC86_S2	NCTC86EC_RS03705	putative colanic acid polymerase WcaD	SNP	nonsyn
NCTC86_S2	NCTC86EC_RS03810	capsular biosynthesis protein	SNP	nonsyn
NCTC86_S2	NCTC86EC_RS25625	transposase	Indel	
NCTC86_S2	NCTC86EC_RS04575	RNA polymerase sigma factor FliA	SNP	nonsyn
NCTC86_S2	NCTC86EC_RS04655	tyrosine transporter TyrP	SNP	nonsyn
NCTC86_S2	NCTC86EC_RS05635	membrane protein	SNP	nonsyn
NCTC86_S2	NCTC86EC_RS06680	glutamate decarboxylase	SNP	nonsyn
NCTC86_S2	NCTC86EC_RS06805	respiratory nitrate reductase 2 alpha chain	SNP	nonsyn
NCTC86_S2	NCTC86EC_RS07275	tRNA 2-thiocytidine(32) synthetase TtcA	Indel	
NCTC86_S2	NCTC86EC_RS07275	tRNA 2-thiocytidine(32) synthetase TtcA	SNP	nonsyn
NCTC86_S2	NCTC86EC_RS07345	methylated-DNA--protein-cysteinemethyltransferase	Indel	
NCTC86_S2	NCTC86EC_RS07505	phage shock protein PspA	SNP	nonsyn
NCTC86_S2	NCTC86EC_RS07655	GTP cyclohydrolase II	SNP	nonsyn
NCTC86_S2	NCTC86EC_RS08270	oligopeptide ABC transporter substrate-binding protein OppA	SNP	nonsyn
NCTC86_S2	NCTC86EC_RS08510	adhesin	SNP	nonsyn
NCTC86_S2	NCTC86EC_RS09055	PTS glucose EIICB component	SNP	nonsyn
NCTC86_S2	NCTC86EC_RS09160	flgI flagellar P-ring protein	SNP	nonsyn

NCTC86_S2	NCTC86EC_RS09600	IS110 family transposase	SNP	nonsyn
NCTC86_S2	NCTC86EC_RS09880	hypothetical protein	SNP	nonsyn
NCTC86_S2	NCTC86EC_RS10015	hypothetical protein	SNP	nonsyn
NCTC86_S2	NCTC86EC_RS10095	dihydroorotate dehydrogenase 2	SNP	nonsyn
NCTC86_S2	NCTC86EC_RS10270	30S ribosomal protein S1	SNP	nonsyn
NCTC86_S2	NCTC86EC_RS10315	formate C-acetyltransferase	SNP	nonsyn
NCTC86_S2	NCTC86EC_RS10360	dimethyl sulfoxide reductase subunit A	SNP	nonsyn
NCTC86_S2	NCTC86EC_RS10530	restriction endonuclease	SNP	nonsense
NCTC86_S2	NCTC86EC_RS10725	malate transporter	SNP	nonsyn
NCTC86_S2	NCTC86EC_RS10745	macrolide ABC transporter ATP-binding protein	Indel	
NCTC86_S2	NCTC86EC_RS10750	hypothetical protein	Indel	
NCTC86_S2	NCTC86EC_RS10755	hypothetical protein	Indel	
NCTC86_S2	NCTC86EC_RS10840	DNA-binding protein	SNP	nonsyn
NCTC86_S2	NCTC86EC_RS11095	hydroxylamine reductase	SNP	nonsyn
NCTC86_S2	NCTC86EC_RS11350	glycyl radical enzyme	SNP	nonsyn
NCTC86_S2	NCTC86EC_RS12035	two-component system sensor histidine kinase KdbD	SNP	nonsyn
NCTC86_S2	NCTC86EC_RS12130	PTS N-acetylglucosamine EIICBA component	SNP	nonsyn
NCTC86_S2	NCTC86EC_RS12495	hypothetical protein	Indel	
NCTC86_S2	NCTC86EC_RS12645	cation transporter	SNP	nonsyn
NCTC86_S2	NCTC86EC_RS12660	cation efflux system protein CusC	SNP	nonsyn
NCTC86_S2	NCTC86EC_RS14015	cyanate transporter	SNP	nonsense
NCTC86_S2	NCTC86EC_RS14050	2-methylcitrate dehydratase	SNP	nonsyn
NCTC86_S2	NCTC86EC_RS14065	propionate catabolism operon regulatory protein PrpR	SNP	nonsyn
NCTC86_S2	NCTC86EC_RS14795	type VI secretion system-associated protein	SNP	nonsyn
NCTC86_S2	NCTC86EC_RS14915	chaperone protein ClpB	SNP	nonsyn
NCTC86_S2	NCTC86EC_RS15000	30S ribosomal protein S16	SNP	nonsyn
NCTC86_S2	NCTC86EC_RS15600	CRISPR-associated helicase/endonuclease Cas3	SNP	nonsyn
NCTC86_S2	NCTC86EC_RS15990	bifunctional 2-acylglycerophosphoethanolamine acyltransferase/acyl-ACP synthetase	SNP	nonsyn
NCTC86_S2	NCTC86EC_RS16450	transcriptional regulator ArgP	SNP	nonsyn
NCTC86_S2	NCTC86EC_RS16585	hypothetical protein	SNP	nonsyn
NCTC86_S2	NCTC86EC_RS17020	restriction endonuclease subunit M	SNP	nonsyn
NCTC86_S2	NCTC86EC_RS17100	polysialic acid transporter	Indel	
NCTC86_S2	NCTC86EC_RS17170	acyl-CoA synthetase	SNP	nonsyn
NCTC86_S2	NCTC86EC_RS17690	transcriptional regulator	SNP	nonsyn
NCTC86_S2	NCTC86EC_RS17790	GntR family transcriptional regulator	SNP	nonsyn
NCTC86_S2	NCTC86EC_RS17840	DUF805 domain-containing protein	Indel	
NCTC86_S2	NCTC86EC_RS18655	efflux transporter periplasmic adaptor subunit	SNP	nonsyn
NCTC86_S2	NCTC86EC_RS18995	general secretory pathway protein	SNP	nonsyn
NCTC86_S2	NCTC86EC_RS19090	glutathione-regulated potassium-efflux system protein KefB	SNP	nonsyn
NCTC86_S2	NCTC86EC_RS19370	two-component system response regulator OmpR	SNP	nonsyn
NCTC86_S2	NCTC86EC_RS19415	Fe-S biogenesis protein NfuA	SNP	nonsyn
NCTC86_S2	NCTC86EC_RS19435	transcriptional regulator MalT	SNP	nonsyn
NCTC86_S2	NCTC86EC_RS20515	RHS element protein	SNP	nonsyn
NCTC86_S2	NCTC86EC_RS20525	hypothetical protein	Indel	
NCTC86_S2	NCTC86EC_RS20965	sulfatase/phosphatase superfamily protein	SNP	nonsyn
NCTC86_S2	NCTC86EC_RS21495	polysaccharide chain length modulation protein	Indel	
NCTC86_S2	NCTC86EC_RS21515	glucose-1-phosphate thymidylyltransferase 2	SNP	nonsyn
NCTC86_S2	NCTC86EC_RS21540	enterobacterial common antigen polymerase	SNP	nonsyn
NCTC86_S2	NCTC86EC_RS21550	transporter	Indel	
NCTC86_S2	NCTC86EC_RS21590	uroporphyrinogen-III C-methyltransferase	SNP	nonsyn
NCTC86_S2	NCTC86EC_RS22790	malG maltose ABC transporter permease	SNP	nonsyn
NCTC86_S2	NCTC86EC_RS22830	hypothetical protein	SNP	nonsyn
NCTC86_S2	NCTC86EC_RS22900	replicative DNA helicase	SNP	nonsyn
NCTC86_S2	NCTC86EC_RS22975	hypothetical protein	SNP	nonsyn
NCTC86_S2	NCTC86EC_RS23145	alpha-D-ribose 1-methylphosphonate 5-phosphate C-P-lyase	SNP	nonsyn
NCTC86_S2	NCTC86EC_RS23240	arginine decarboxylase	SNP	nonsyn
NCTC86_S2	NCTC86EC_RS23450	nucleoside permease	Indel	
NCTC86_S2	NCTC86EC_RS24705	fec operon regulator FecR	SNP	nonsyn
NCTC86_S2	NCTC86EC_RS24780	hypothetical protein	Indel	
NCTC86_S2	NCTC86EC_RS24805	hypothetical protein	SNP	nonsyn
NCTC86_S2	NCTC86EC_RS25300	trifunctional nicotinamide-nucleotide adenylyltransferase/ribosylnicotinamide kinase/transcriptional regulator NadR	Indel	
NCTC86_S3	NCTC86EC_RS00330	ribulokinase	SNP	nonsyn
NCTC86_S3	NCTC86EC_RS00530	cell division protein ZapD	Indel	
NCTC86_S3	NCTC86EC_RS00540	guanosine monophosphate reductase	SNP	nonsyn
NCTC86_S3	NCTC86EC_RS00670	hypothetical protein	SNP	nonsyn
NCTC86_S3	NCTC86EC_RS01420	hypothetical protein	SNP	nonsyn
NCTC86_S3	NCTC86EC_RS01440	cytoskeleton protein RodZ	SNP	nonsyn
NCTC86_S3	NCTC86EC_RS01450	histidine--tRNA ligase	SNP	nonsyn
NCTC86_S3	NCTC86EC_RS02635	o-succinylbenzoate synthase	Indel	
NCTC86_S3	NCTC86EC_RS03800	hypothetical protein	SNP	nonsyn
NCTC86_S3	NCTC86EC_RS03850	imidazole glycerol phosphate synthase cyclase subunit	SNP	nonsyn
NCTC86_S3	NCTC86EC_RS03880	ATP phosphoribosyltransferase	SNP	nonsyn
NCTC86_S3	NCTC86EC_RS25625	transposase	Indel	
NCTC86_S3	NCTC86EC_RS05315	PrkA family serine protein kinase	SNP	nonsyn
NCTC86_S3	NCTC86EC_RS05385	MFS transporter	Indel	
NCTC86_S3	NCTC86EC_RS05385	MFS transporter	SNP	nonsyn
NCTC86_S3	NCTC86EC_RS05625	hypothetical protein	SNP	nonsyn
NCTC86_S3	NCTC86EC_RS06185	TetR family transcriptional regulator	SNP	nonsyn
NCTC86_S3	NCTC86EC_RS07575	peptide transport system ATP-binding protein SapD	SNP	nonsyn
NCTC86_S3	NCTC86EC_RS09170	flagellar basal body rod protein FlgG	SNP	nonsyn
NCTC86_S3	NCTC86EC_RS09770	transporter	SNP	nonsyn
NCTC86_S3	NCTC86EC_RS10745	macrolide ABC transporter ATP-binding protein	Indel	
NCTC86_S3	NCTC86EC_RS10750	hypothetical protein	Indel	
NCTC86_S3	NCTC86EC_RS10755	hypothetical protein	Indel	
NCTC86_S3	NCTC86EC_RS11115	NAD(P)-dependent oxidoreductase	SNP	nonsyn
NCTC86_S3	NCTC86EC_RS11520	EEP domain-containing protein	SNP	nonsyn
NCTC86_S3	NCTC86EC_RS11700	phospho-2-dehydro-3-deoxyheptonate aldolase Phe-sensitive	SNP	nonsyn
NCTC86_S3	NCTC86EC_RS12600	IS4 family transposase	Indel	
NCTC86_S3	NCTC86EC_RS12655	cation efflux system protein CusF	SNP	nonsyn
NCTC86_S3	NCTC86EC_RS13075	DUF1116 domain-containing protein	SNP	nonsyn
NCTC86_S3	NCTC86EC_RS13160	hypothetical protein	Indel	
NCTC86_S3	NCTC86EC_RS13845	D-alanine--D-alanine ligase A	SNP	nonsyn
NCTC86_S3	NCTC86EC_RS14790	ClpV1 family T6SS ATPase	SNP	nonsyn
NCTC86_S3	NCTC86EC_RS17095	ABC transporter ATP-binding protein	SNP	nonsyn
NCTC86_S3	NCTC86EC_RS17690	transcriptional regulator	SNP	nonsyn
NCTC86_S3	NCTC86EC_RS19365	two-component sensor histidine kinase	SNP	nonsyn
NCTC86_S3	NCTC86EC_RS20280	autotransporter beta-domain protein	Indel	
NCTC86_S3	NCTC86EC_RS20525	hypothetical protein	Indel	
NCTC86_S3	NCTC86EC_RS20620	hypothetical protein	SNP	nonsyn
NCTC86_S3	NCTC86EC_RS20840	hypothetical protein	Indel	
NCTC86_S3	NCTC86EC_RS22210	universal stress protein D	SNP	nonsyn
NCTC86_S3	NCTC86EC_RS22830	hypothetical protein	SNP	nonsyn
NCTC86_S3	NCTC86EC_RS25355	cell envelope integrity protein CreD	SNP	nonsyn
