# Supplementary material for: The Odyssey of the Ancestral Escherich Strain through Culture Collections: an Example of Allopatric Diversification
Source: mSphere. 2018 Jan 31;3(1):e00553-17. doi: 10.1128/mSphere.00553-17 (PMC5793043; doi:10.1128/mSphere.00553-17)
Supplement: TABLE S7 [file sph001182464st7.pdf]

TABLE S7. Mutations in *ompR* and *envZ at the protein level,* prediction of their effect and frequencies in Uniprot database

Genes	Gene products	Mutations at the protein level*	Predictions of functional effect			Frequency
			SIFT	Polyphen	PROVEAN	
ompR	Transcriptional regulatory protein OmpR	M40R	0.10	Possibly damaging (0.836)	Deleterious (-5,249)	0/115
		R190C	0.24	Possibly damaging (0.922)	Deleterious (-6,483)	0/115
		Y102H	0.00 (low confidence)	Probably damaging (1.000)	Deleterious (-4,98)	0/115
envZ	Osmolarity sensor protein EnvZ	V227A	0.02	Benign (0.279)	Deleterious (-3,529)	0/121

*Mutations predicted to be deleterious at the protein level at least with two of the three softwares and absent in Uniprot database are highlighted in red
