# Supplementary material for: The Odyssey of the Ancestral Escherich Strain through Culture Collections: an Example of Allopatric Diversification
Source: mSphere. 2018 Jan 31;3(1):e00553-17. doi: 10.1128/mSphere.00553-17 (PMC5793043; doi:10.1128/mSphere.00553-17)
Supplement: TABLE S8 [file sph001182464st8.pdf]

TABLE S8. Minimum Inhibitory Concentration (mg/L) values of antibiotics for squatter colonies

Antibiotics	NCTC86_squatter1	NCTC86_squatter2	NCTC86_squatter3	ATCC4157_squatter1	ATCC4157_squatter2	ATCC4157_squatter3	Breakpoints*	HS(<=1%)£	VS(<=5%)£
benzylpenicillin	>32	>32	>32	12	8	12	NC§	-	-
amoxicillin	1.5	1.5	1.5	3	3	3	8	0.5	1
cefotaxim	0.016	0.016	0.008	0.023	0.032	0.032	1	0.008	0.016
imipenem	0.064	0.047	0.094	0.064	0.064	0.047	2	0.016	0.032
ertapenem	<0,002	<0,002	0.002	0.002	0.004	0.004	0.5	ND€	ND€
chloramphenicol	1.5	1	0.75	1	1	1	8	1	ND€
tetracyclin	0.125	0.5	0.19	0.5	0.19	0.25	8	0.25	0.5
colistin	0.016	0.016	0.016	0.016	<0,016	0.016	2	0.064	0.125
ciprofloxacin	<0,002	<0,002	<0,002	<0,002	<0,002	<0,002	0.25	0.002	0.004
gentamicin	0.19	0.19	0.25	0.25	0.125	0.25	2	0.125	ND€
cotrimoxazol	0.003	0.003	0.012	0.016	0.016	0.016	2	0.016	ND€

*Breakpoints are clinical breakpoints from the CASFM-EUCAST (V1.0 2017/03), or Epidemiological Cut-OFF (£) from EUCAST if clinical breakpoint unavailable

£Using the MIC distribution from EUCAST we defined hypersusceptible (HS) (green) and Very-susceptible (VS) (blue) cut-off as the lowest MIC encompassing 1 and 5% of the E. coli strains, respectively

§NC : no cut-off (clinical or epidemiological) provided by EUCAST

€ND : not determined
